# Detection of *Bacillus anthracis* DNA in Complex Soil and Air Samples Using Next-Generation Sequencing

**DOI:** 10.1371/journal.pone.0073455

**Published:** 2013-09-09

**Authors:** Nicholas A. Be, James B. Thissen, Shea N. Gardner, Kevin S. McLoughlin, Viacheslav Y. Fofanov, Heather Koshinsky, Sally R. Ellingson, Thomas S. Brettin, Paul J. Jackson, Crystal J. Jaing

**Affiliations:** 1 Physical and Life Sciences, Lawrence Livermore National Laboratory, Livermore, California, United States of America; 2 Global Security Directorates, Lawrence Livermore National Laboratory, Livermore, California, United States of America; 3 Eureka Genomics, Hercules, California, United States of America; 4 Department of Genome Science and Technology, University of Tennessee, Knoxville, Tennessee, United States of America; 5 Oak Ridge National Laboratory, Oak Ridge, Tennessee, United States of America; Loyola University Medical Center, United States of America

## Abstract

*Bacillus anthracis* is the potentially lethal etiologic agent of anthrax disease, and is a significant concern in the realm of biodefense. One of the cornerstones of an effective biodefense strategy is the ability to detect infectious agents with a high degree of sensitivity and specificity in the context of a complex sample background. The nature of the *B. anthracis* genome, however, renders specific detection difficult, due to close homology with *B. cereus* and *B. thuringiensis*. We therefore elected to determine the efficacy of next-generation sequencing analysis and microarrays for detection of *B. anthracis* in an environmental background. We applied next-generation sequencing to titrated genome copy numbers of *B. anthracis* in the presence of background nucleic acid extracted from aerosol and soil samples. We found next-generation sequencing to be capable of detecting as few as 10 genomic equivalents of *B. anthracis* DNA per nanogram of background nucleic acid. Detection was accomplished by mapping reads to either a defined subset of reference genomes or to the full GenBank database. Moreover, sequence data obtained from *B. anthracis* could be reliably distinguished from sequence data mapping to either *B. cereus* or *B. thuringiensis*. We also demonstrated the efficacy of a microbial census microarray in detecting *B. anthracis* in the same samples, representing a cost-effective and high-throughput approach, complementary to next-generation sequencing. Our results, in combination with the capacity of sequencing for providing insights into the genomic characteristics of complex and novel organisms, suggest that these platforms should be considered important components of a biosurveillance strategy.

## Introduction

*Bacillus anthracis* is the gram-positive etiologic agent responsible for the potentially fatal infectious disease anthrax. This species can form dormant endospores resistant to extreme environmental conditions and can persist for long periods in terrestrial or aquatic environments. Spores function as infectious agents by exiting the dormant state upon contact with a nutrient-rich environment, which, in humans, occurs following respiratory, gastrointestinal, or cutaneous exposure. This germination process subsequently leads to growth in host tissues and, later, expression of toxins responsible for deleterious physiological effects in humans and other mammals [1]. These toxins, along with factors required for bacterial encapsulation, are encoded on two plasmids, pXO1 and pXO2 [2]. Two of the anthrax toxin proteins, edema factor (EF) and lethal factor (LF), each form binary complexes with protective antigen (PA), causing severe edema and cell death [3].

The acute and potentially lethal nature of aerosol infection by *B. anthracis*, in combination with the resilience of its spores upon exposure to heat and radiation, contributes significantly toward the possibility of this pathogen being used as an aerosolized bioweapon. During the months of October to November of 2001, *B. anthracis* spores were sent via public mail to government and news organizations, resulting in 22 cases of anthrax and 5 deaths [4]. In the decade following these attacks there has been an increased interest and investment in surveillance to detect bioterrorism agents, particularly *B. anthracis*, in complex samples.

One of the most important aspects of an effective response to a biologically-based attack is the ability to detect potentially infectious agents with a high degree of sensitivity and specificity within a complex mixture. The LD_50_ for inhalation anthrax is estimated to be approximately 8,000 colony-forming units (CFU), although the minimal infectious dose is postulated to be much lower [5]. Successful detection technologies will therefore need to be capable of identifying low quantities of *B. anthracis* bacilli. An additional major challenge to *B. anthracis* detection lies in its close kinship with *B. thuringiensis* and *B. cereus*, two common environmental species. The high degree of sequence similarity among these three species and the potential for horizontal plasmid transfer pose significant challenges when attempting to distinguish closely related species within this genus.

A number of methods, ranging from basic to complex in their application, have been employed for detecting *B. anthracis*. Most simply, the bacilli may be cultured and identified on blood agar. Microbiological techniques are, however, slow and require personnel trained in recognizing bacterial morphology. Moreover, while culture methods can provide extensive information about a microbe, these procedures are often lengthy and do not provide a genetic signature for the isolate being characterized. Identification based on biochemical characteristics has been examined, including assays for lipids characteristic of certain bacilli [6] and intact cell mass spectrometry for the construction of a spectrum typifying *B. anthracis* [7]. Such methods, however, often require pure cultures, specific growth conditions, and depend on the quality of available lipid and proteomic databases [8]. Numerous immunoassays have been developed to detect *B. anthracis* antigens and toxins [9–11], but their utility is limited by low sensitivity and reduced specificity due to significant antigenic homology between *B. anthracis* and some *B. cereus* isolates [11]. The current gold standard for detection and quantification of bacterial species in environmental samples is real-time quantitative PCR. Numerous such assays have been evaluated and tested for detection of *B. anthracis* [12–14]. Advantages of this approach include relatively high sensitivity and a protocol performed in most standard laboratories. These assays do not, however, provide in depth genomic detail on the strain detected, and may suffer from non-specific detection of near-neighbor species. All of the above techniques currently in place have limited potential for characterization of novel or previously uncharacterized isolates.

The increased capacity and reduced cost of next-generation DNA sequencing have resulted in its increased application toward microbial identification and characterization. The ability to rapidly sequence full bacterial genomes provides the opportunity for heightened specificity when comparing near genetic neighbors, and for identifying rare or novel variants. One effective sequencing strategy that is commonly employed involves targeting of specific bacterial amplicons present across multiple species. In targeted approaches, the 16S small ribosomal subunit is often used due to its broad applicability to bacterial species and relative evolutionary conservation. 16S ribosomal DNA (rDNA) sequencing yields high-level taxonomic data regarding the composition of a microbial community. It is not, however, likely to yield strain or species-specific resolution [15]. Additionally, 16S rDNA-based methods will not identify bacterial plasmids, or distinguish between samples which do or do not contain plasmid DNA, which is particularly relevant in the case of the pXO1 and pXO2 plasmids of *B. anthracis*. Finally, instances may arise when it would be useful to attain comprehensive detection of both bacterial and non-bacterial organisms in conjunction, including fungi, parasites, DNA viruses, and some RNA viruses. Performing shotgun metagenomic sequencing, paired with specialized bioinformatics analyses, has the potential for making distinctions at a higher resolution, as well as for identifying non-prokaryotic species. Due to our interest in identifying highly similar *Bacillus* strains with enhanced specificity in environmental samples, we employed the shotgun metagenomic approach.

We added defined copy numbers of the *B. anthracis* genome to whole nucleic acid extracted from aerosol and soil particulates, simulating a complex environmental background, to assess the efficacy of whole genome next-generation sequencing for detection of *B. anthracis* in the environment. We sequenced this material using the Illumina and 454 platforms and processed the resultant data using multiple bioinformatics approaches. Finally, we processed the same *B. anthracis*-spiked samples using a previously developed microbial census microarray to compare the sensitivity of this more cost-effective and higher-throughput approach to next-generation sequencing.

## Materials and Methods

### DNA extraction from environmental samples

Soil was collected in the downtown areas of Oakland and San Francisco, CA. Four samples were collected at four different sites in each city. No specific permissions were required for soil sample acquisition in the field, as all collections were performed on public, non-protected land. These field studies did not involve any endangered or protected species. DNA was extracted from soil using the UltraClean Soil DNA Isolation Kit (MoBio, Carlsbad, CA) using the manufacturer’s alternative protocol for maximum yield. Following extraction, 1 ng of each extracted DNA was used in a real-time PCR assay to test for inhibition. All samples showed a high level of PCR inhibition and were reprocessed starting from Step 12 of the MoBio alternative protocol, intended to remove excess humic acid.

For aerosol samples, primary sampling filters were obtained from BioWatch aerosol collection units (collected April 2009) from the National Capital Region Laboratory. Filters were added to 30 mL 100 mM phosphate buffer (pH 7.4) containing 0.05% (v/v) Tween 80 and vortexed for 30 seconds followed by incubation on a rocking shaker for 15 minutes. This agitation procedure was repeated three times. The filters were removed from the tube and the remaining solution centrifuged at 3200 x g for 30 minutes at 4^o^C. Following centrifugation, the supernatant was removed and discarded. DNA purification was subsequently performed using the UltraClean Soil DNA Isolation Kit (MoBio). DNA concentrations were measured using a Qubit fluorometer (Life Technologies, Grand Island, NY).

### Addition of *Bacillus anthracis* Ames DNA to environmental samples

*B. anthracis* Ames DNA was acquired from the LLNL select agent laboratory and confirmed to be free of viable cells or spores by plating of 1/10 the total DNA volume on blood agar plates followed by incubation at 37°C for 72 hours. DNA concentration was determined by measurement using a Qubit fluorometer and the number of femtograms/genome equivalent was determined based on GenBank chromosomal and plasmid genome sizes. *B. anthracis* genomic DNA was added to nucleic acid extracted from the two environmental sources in six amounts (1, 10, 100, 1000, 10000, and 100000 genomic equivalents of *B. anthracis* DNA). *B. anthracis* DNA was mixed with 100 pg of DNA extracted from aerosol filters or 1 ng DNA extracted from the combined Oakland and San Francisco soil extracts.

### Whole genome amplification and purification

All of the above *B. anthracis*-spiked samples were amplified using the REPLI-g Midi Kit (Qiagen, Gaithersburg, MD), intended to provide uniform whole genome amplification. This kit was used according to the manufacturer’s instructions, allowing samples to amplify for 16 hours at 30^°^C. Amplified samples were purified using the Qiaquick PCR Purification Kit (Qiagen), and yielded 17.0 ± 1.8 µg amplified DNA for each sample.

### Illumina and 454 sequence generation

Amplified samples containing 1, 10, 100, 1000, 10000, or 100000 genome equivalents of the *B. anthracis* genome were provided to Eureka Genomics for library preparation and sequence data generation using the Illumina platform. A paired end non-indexed standard Illumina library was prepared for each sample and sequence reads generated on the Illumina GAIIx, running one sample per lane. Soil samples were sequenced using 51 cycles of paired end reads, while aerosol samples were sequenced using 51 cycles of single end reads. For the purpose of analysis, the paired end reads were decoupled and used as if single reads were generated. As a result, soil samples exhibit roughly double the number of sequence reads (decoupled singletons), although the number of independent samplings is not doubled. Since the paired end read is typically generated from a single sequence fragment, the sensitivity of the soil samples, in terms of the ability to detect rare environmental organisms, is not significantly increased when compared to aerosol samples.

The same samples sequenced using Illumina technology were also processed via 454 pyrosequencing. Based on results from Illumina sequence analysis and anticipated longer reads, we selected a more narrow range of samples that would still provide a sufficiently dynamic range of response and added one sample containing zero genomic copies of *B. anthracis*. Amplified, *B. anthracis*-spiked samples containing 0, 1, 10, or 100 genome equivalents in either aerosol or soil backgrounds were provided to the DNA Sequencing Center at Brigham Young University (BYU). 454 sequence reads were generated from one 96-well plate on the Genome Sequencer FLX using recommended protocols (454 Life Sciences, Branford, CT).

All sequence data are available via the Sequence Read Archive (SRA) database (accession number SRP025362).

### Illumina and 454 sequence mapping and data analysis

Eureka Genomics performed analysis on reads generated by Illumina sequencing. In all cases, paired end reads were decoupled prior to analysis and searched/aligned separately. This was done to maximize sensitivity of the alignment, to remove ambiguity associated with treating broken read pairs (where only one portion of the read pair maps to an organism), and to make search and alignment results more consistent with 454 sequence read analysis (single-end). Publicly available Burrows-Wheeler Aligner software (default parameters, version 0.6.2-r126) was used for mapping of Illumina reads to specified background and target reference genomes. Publicly available bowtie software (default parameters, version 0.12.9) was used for search and alignment of Illumina-generated shotgun metagenomic sequence data against all sequences present in NCBI GenBank (current as of April 2013). The length of Illumina GAIIx reads (51 bp) resulted in a high likelihood of multiple optimal alignments in different genomes. As such, a MegaBlast top-hit only approach, used for the analysis of 454 reads, carries an amplified risk of producing an excessively high false positive hit rate, and is prohibitively computationally expensive. All hits up to three mismatches were kept. The resulting output was parsed from each bowtie run to obtain taxonomy IDs (taxID) matched by each read. All possible hits for each read were recorded and classified using NCBI taxonomy classification schema.

A possible source of bias involved the fact that multiple substrain reference genomes can artificially inflate the number of mapped reads for a given species. Each read was counted as matching to a given taxID only once to avoid such bias and to preclude inflation associated with tandem repeats. Multiple bacterial strain sequences were also “collapsed” such that reads mapping to substrains were instead counted as mapping to a single parent species. This is particularly helpful when trying to distinguish between reads mapping to closely related species that share significant sequence similarity, since mapping to multiple substrains of a given bacterial species could erroneously suggest that a species that is present in abundance in a sample is present at only low concentrations.

Oak Ridge National Laboratory (ORNL) performed analysis of the 454 sequence reads generated by BYU. The vendor-provided gsMapper software (Roche Applied Science, Indianapolis, IN) was used to map 454 reads to corresponding reference sequences using a 98% minimum identity cutoff. The MegaBlast program from NCBI version 2.2.18 was used with nucleotide and taxonomy databases current as of December 2010 for the whole microbiome analysis of 454 reads. Each input file was split into 256 smaller files, using each of these smaller files as input for MegaBlast, running on a local Linux cluster. The resulting output from each MegaBlast run was parsed to obtain the best hit NCBI sequence identification and corresponding bacterial species for each queried sequence.

### Census microarray probe design

All available bacterial and viral genome and fragment sequences were downloaded from NCBI GenBank, the Joint Genome Institute, the Integrated Microbial Genomics and Comprehensive Microbial Genomics projects at the J. Craig Venter Institute, the Sanger Institute, and the San Francisco Blood Systems Research Institute. Sequence data for complete genomes, viral segments, and plasmids were current as of August 2009, and for sequence fragments as of January 2009. The probe design process began by identifying family-specific sequence regions. The number of families, species, genomes, and sequences referenced for probe design are given in Table S7. In total, sequences representing 5,719 viruses and 14,765 bacterial species (including incomplete sequence fragments) were employed for array design. These sequences correspond to 80 viral families and 274 bacterial families. Sequences from each target family with perfect matches to sequences outside the target family were eliminated. Using the suffix array software Vmatch [16], perfect match subsequences of at least 17 nucleotides long present in non-target viral families or 25 nucleotides long present in the human genome or non-target bacterial families were also eliminated from consideration as possible probe subsequences.

Probes 50-66 bases long were designed for each of the family-specific regions using previously described methods [17]. Briefly, candidate probes were designed using Primer3 [18], followed by prediction of T_m_ and homodimer, hairpin, and probe-target free energy (ΔG) using Unafold [19]. Candidate probes with unsuitable ΔG or T_m_ were excluded as described previously [17]. The range for these parameters included length of 50-66 bp, T_m_ ≥ 80^◦^C, GC% 25-75%, ΔG of homodimer formation > 15 kcal/mol, ΔG of hairpin formation > -11 kcal/mol, and ΔG_adjusted_ (Δ*G*_*complement*_ -1.45ΔG_*hairpin*_ - 0.33ΔG_*homodimer*_) ≤ -52 kcal/mol. An additional minimum sequence complexity constraint was enforced, requiring a trimer frequency entropy of at least 4.5. In the event that an insufficient number of candidate probes per target sequence passed all criteria, parameters (first Unafold and then Primer3) were relaxed to allow an adequate number of probes per target (5-30 detection probes and 1-10 census probes). The candidate probe sequences were then examined via BLAST against the target sequences from which they were designed. Resultant alignments were used to identify which sequences should be represented by each candidate. A target was considered to be represented if it matched a probe with > 85% sequence similarity over the total probe length, with a 29 contiguous base perfect match spanning the central probe base. Probe candidates were then ranked by conservation level.

Separate strategies were employed for detection and census probes. Detection probes were selected as previously described [20]. Essentially, probes corresponding to a higher number of targets in the family were chosen preferentially. Conversely, for census probes, probes detecting fewer targets in the family were preferentially chosen. A secondary dispersal ranking was used to favor probes with genomic loci distant from those that had already been selected to represent the target. 1,235 random negative control sequences, with matched length and GC content, were also included. These controls had no appreciable homology to known sequences based on BLAST similarity, and were used to assess background hybridization intensity. Probes were designed to accommodate the NimbleGen 385K format (Roche). Probe sequences corresponding to organisms identified and referenced in this study have been deposited with the Gene Expression Omnibus (GEO) database (accession number GSE48316).

### Microarray hybridization and data analysis

Amplified environmental DNA spiked with *B. anthracis* Ames genomic DNA was fluorescently labeled using the NimbleGen One-Color DNA Labeling Kit (Roche) according to the manufacturer’s recommended protocols. DNA was purified and hybridized to the census array using the NimbleGen hybridization kit. Samples were allowed to hybridize for 17 hours and washed using the NimbleGen Wash Buffer Kit (Roche). Microarrays were scanned on an Axon GenePix 4000B 5 µm scanner (Molecular Devices, Sunnyvale, CA). Image files were aligned using NimbleScan (Version 2.4) software, and pair text files were exported for data analysis. A previously described maximum likelihood analysis method was used to analyze the microbial hits from samples hybridized to the array [20].

## Results

### Detection of *B. anthracis* in environmental samples by Illumina sequencing and mapping to reference subsets

DNA was extracted from both aerosol filter particulates and soil to create a simulated nucleic acid background. In order to evaluate the ability of genomic platforms to identify and distinguish *B. anthracis* DNA within a complex nucleic acid mixture, *B. anthracis* genomic DNA was spiked at six concentrations into either 100 pg aerosol DNA or 1 ng soil DNA. Two different quantities were used to reflect the likely increased background density in soil samples. Environmental samples, however, will likely contain a broad range of bacterial, fungal, plant, and animal DNA, thus it is difficult to estimate bacterial load based upon total DNA quantity. Genomic DNA was used for spiked-in samples instead of whole bacilli or spores due to select agent limitations in the genomics laboratories where this study was performed. All samples were subjected to whole genome amplification (WGA) followed by Illumina sequencing (Table 1).

**Table 1:** Genome equivalents of *B. anthracis* DNA added to environmental background DNA for detection assessment.

***Aerosolbackground***						
*B. anthracis* genome copies*	100,000	10,000	1,000	100	10	1
**Amount *B. anthracis* DNA**	560 pg	56 pg	5.6 pg	560 fg	56 fg	5.6 fg
**Amount aerosol filter DNA**	100 pg	100 pg	100 pg	100 pg	100 pg	100 pg
**% *B. anthracis* DNA in aerosol DNA**	84.85%	35.90%	5.30%	0.56%	0.060%	0.006%
***Soilbackground***						
*B. anthracis* genome copies*	100,000	10,000	1,000	100	10	1
**Amount *B. anthracis* DNA**	560 pg	56 pg	5.6 pg	560 fg	56 fg	5.6 fg
**Amount soil DNA**	1 ng	1 ng	1 ng	1 ng	1 ng	1 ng
**% *B. anthracis* DNA in soil DNA**	35.90%	5.30%	0.56%	0.060%	0.006%	0.001%

* The number of genome equivalents was calculated as 5.6 femtograms based on a chromosome and the two plasmids pXO1 and pXO2 as one genome equivalent. Calculations were based on the published genome and plasmid sizes in base pairs using accession numbers NC_007530 (chromosome), NC_007322 (pXO1), and NC_007323 (pXO2).

We identified *B. anthracis* DNA by mapping sequence reads to a defined subset of target reference genomes that included the *B. anthracis* Ames complete genome (accession number NC_007530) and the pXO1 and pXO2 virulence plasmids (accession numbers NC_007322 and NC_007323 respectively). The background reference subset included eleven finished genomes chosen to represent nucleic acid background and evaluate specificity and sample variation (Table 2). Several of these genomes represent other organisms of interest from a biodefense perspective, such as *Francisella tularensis* and *Burkholderia pseudomallei*. These were selected to confirm that sequence reads corresponding to *B. anthracis* would not map to these biothreat agents. Also included were several organisms of clinical or environmental interest to serve as additional controls for non-specific alignment of *B. anthracis* sequence reads. Additional analyses later in this study will consider the same sequence data aligned to the full NCBI GenBank database.

**Table 2:** Bacterial reference genomes used for mapping of Illumina and 454 sequencing reads.

Target Reference Genomes	Accession No.
***Bacillus anthracis* str. Ames, complete genome**	NC_007530
***Bacillus anthracis* virulence plasmid pXO1, complete sequence**	NC_007322
***Bacillus anthracis* plasmid pXO2, complete sequence**	NC_007323
*Bacillus anthracis* str. Sterne, complete genome*	NC_005945
*Bacillus anthracis* str. 'Ames Ancestor' plasmid pXO1, complete sequence*	NC_007322
*Bacillus anthracis* str. 'Ames Ancestor' plasmid pXO2, complete sequence*	NC_007323
***Bacillus thuringiensis* str. Al Hakam, complete genome**	NC_008600
***Bacillus thuringiensis* str. Al Hakam, plasmid pALH1, complete sequence**	NC_008598
*Bacillus cereus* biovar *anthracis* str. CI, complete genome**	NC_014335
*Bacillus cereus* biovar *anthracis* str. CI plasmid pCI-XO1, complete sequence**	NC_014331
*Bacillus cereus* biovar *anthracis* str. CI plasmid pCI-XO2, complete sequence**	NC_014332
*Bacillus cereus* biovar *anthracis* str. CI plasmid pBAslCI14, complete sequence**	NC_014333
**Background Reference Genomes**	**Accession No.**
***Burkholderia pseudomallei* strain K96243, chromosome 1, complete sequence**	NC_006350
***Escherichia coli* O157:H7 EDL933, complete genome**	NC_002655
***Francisellatularensis* subsp. *tularensis* SCHU S4 complete genome**	NC_006570
***Pseudomonas aeruginosa* PAO1, complete genome**	NC_002516
***Rhodopseudomonas palustris* CGA009 complete genome**	NC_005296
***Sinorhizobium meliloti* 1021 complete chromosome**	NC_003047
***Staphylococcus aureus* subsp. *aureus* N315 DNA, complete genome**	NC_002745
***Streptomyces coelicolor* A3 (2) complete genome**	NC_003888
***Yersinia pestis* CO92 complete genome**	NC_003143
***Bacillus subtilis* subsp. *subtilis* str. 168 complete genome**	NC_000964
***Clostridium botulinum* A str. Hall, complete genome**	NC_009698

* Used as reference for 454 sequencing reads only; ** Used as reference for Illumina sequencing reads only. *B. anthracis* target reference genomes were used for identification of reads corresponding to *B. anthracis* in each sample. *B. cereus* and *B. thuringiensis* target reference genomes were used for assessment of specificity provided by the read mapping strategy.

**Figure 1:**
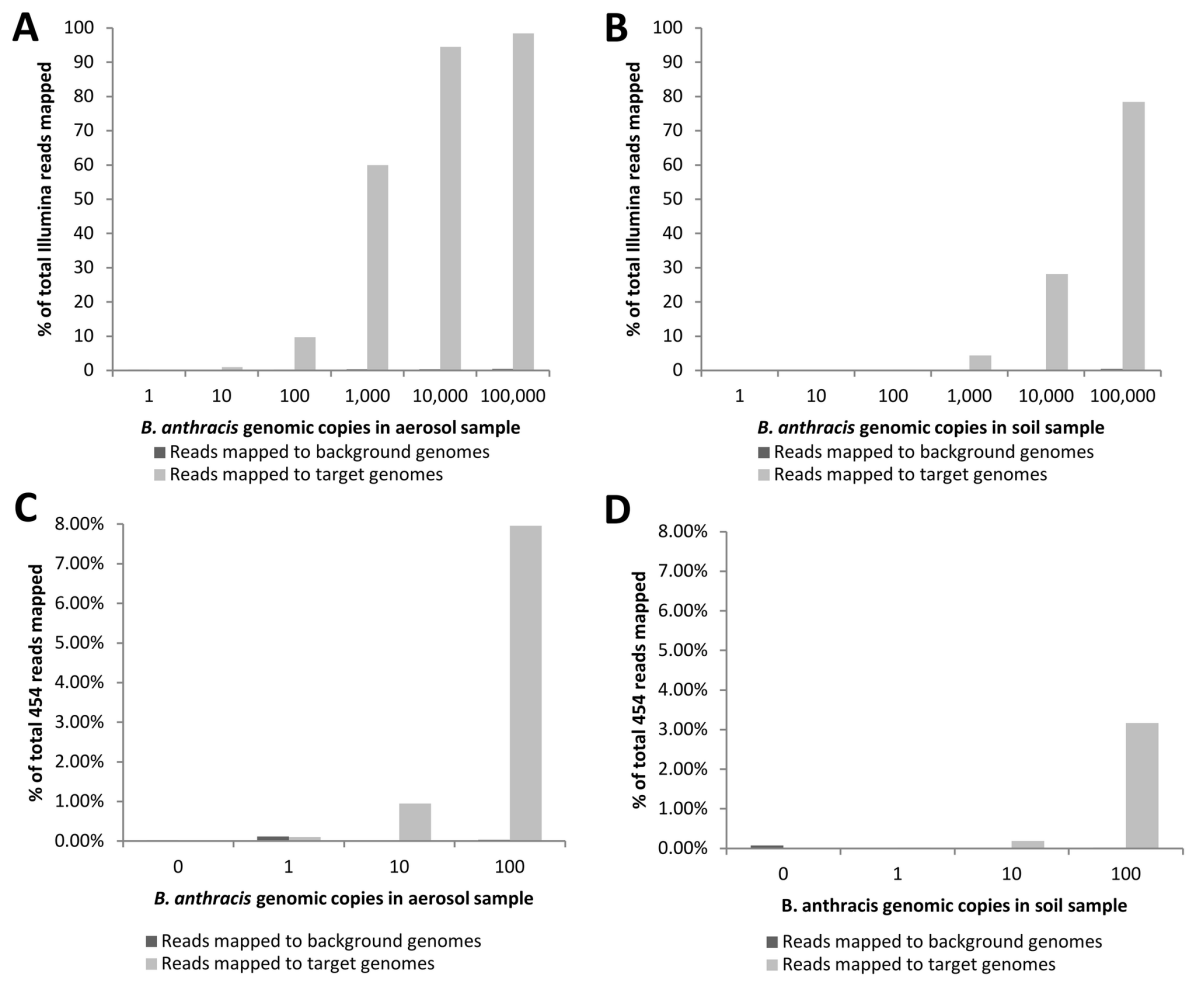
Mapping of sequencing reads obtained from *B. anthracis*-spiked environmental samples to specified reference genomes. *B. anthracis* Ames genomic DNA was combined with background nucleic acid extracted from either aerosol or soil-based material. Increasing genome copy numbers were spiked into samples at 10-fold concentration intervals. Samples were then subjected to whole genome amplification and next-generation sequencing. The resultant reads were mapped to either a target set (*B. anthracis* and plasmids) or a background set of DNA sequences, intended to assess non-specific alignment of *B. anthracis*-derived sequence reads to other genomes. The numbers of reads mapped were normalized to total reads obtained for each sample to standardize results. Shown are the percentage of reads mapped for **A**. Illumina reads from an aerosol background spiked with *B. anthracis* genomic DNA, **B**. Illumina reads from a soil background spiked with *B. anthracis* DNA, **C**. 454 reads from an aerosol background spiked with *B. anthracis* DNA, and **D**. 454 reads from a soil background spiked with *B. anthracis* DNA.

The relative number of reads mapping to the target and background reference genomes using *B. anthracis*-spiked aerosol and soil samples were normalized to the total number of reads obtained for each sample (Figure 1A-B). Absolute read numbers are shown in Figure S1A-B. The relative number of Illumina reads in each aerosol sample mapping to the *B. anthracis* chromosome and plasmids increased from the samples containing one genomic copy to the samples containing 100,000 genomic copies (Figure 1A). There was no appreciable increase in the number of corresponding hits to genomes from the subset of background species.

Detection of an organism in an environmental sample via sequencing requires that sequence reads mapping to the target genome be distinguished from those mapping spuriously to background genomes. Effective detection therefore requires that the normalized number of reads mapping to *B. anthracis* exceeds those mapping to background reference genomes. In aerosol samples, the number of reads mapping to *B. anthracis* was greater than those mapping to background genomes when as few as 10 genomic equivalents of DNA were added to 100 pg of aerosol DNA. In soil samples the percentage of reads mapping to *B. anthracis* exceeded those mapping to background genomes when as few as 100 genomic equivalents were added to 1 ng of soil DNA (Figure 1B). These results demonstrate detection of 100 genome copies (0.56 pg DNA) per 1 ng aerosol or soil-derived DNA. In order to facilitate internal comparison of the methods within this study, detection will be expressed as genome copy number per nanogram of environmental nucleic acid.

It should be noted that an observation of the number of reads mapping to target genomes exceeding those mapping to background genomes is not by itself sufficient for declaring positive detection of a target organism, and that specificity of read mapping to the target genome should also be evaluated. Additionally, alignment to genomes outside the background reference set could alter observed results. The above issues are evaluated in the following studies.

### Parallel detection of *B. anthracis* in environmental samples by 454 sequencing

The *B. anthracis*-spiked samples were also processed via 454 pyrosequencing, and the generated sequence data was aligned to reference genomes as previously (Table 2). Based on the anticipated longer reads obtained by 454 pyrosequencing, we selected a lower quantity of samples which would still provide a sufficiently dynamic range of response. A sample containing zero genomic copies of *B. anthracis* was also added. The percentage of reads mapping to *B. anthracis* from aerosol and soil samples increased with addition of increasing genomic equivalents without an observable increase in the number of matches to the selected background species (Figure 1C-D). Absolute read quantities are given in Figure S1C-D. In aerosol samples, the number of reads mapping to *B. anthracis* was greater than those mapping to background genomes with 10 or more genomic equivalents of DNA in 100 pg of aerosol DNA. In soil samples, the percentage of reads mapping to *B. anthracis* exceeded those mapping to background genomes with 10 or more genomic equivalents in 1 ng of soil DNA. These results indicate 454 sequencing detection of 100 *B. anthracis* genome equivalents per nanogram aerosol background DNA and 10 *B. anthracis* genome equivalents per nanogram soil background DNA.

**Figure 2:**
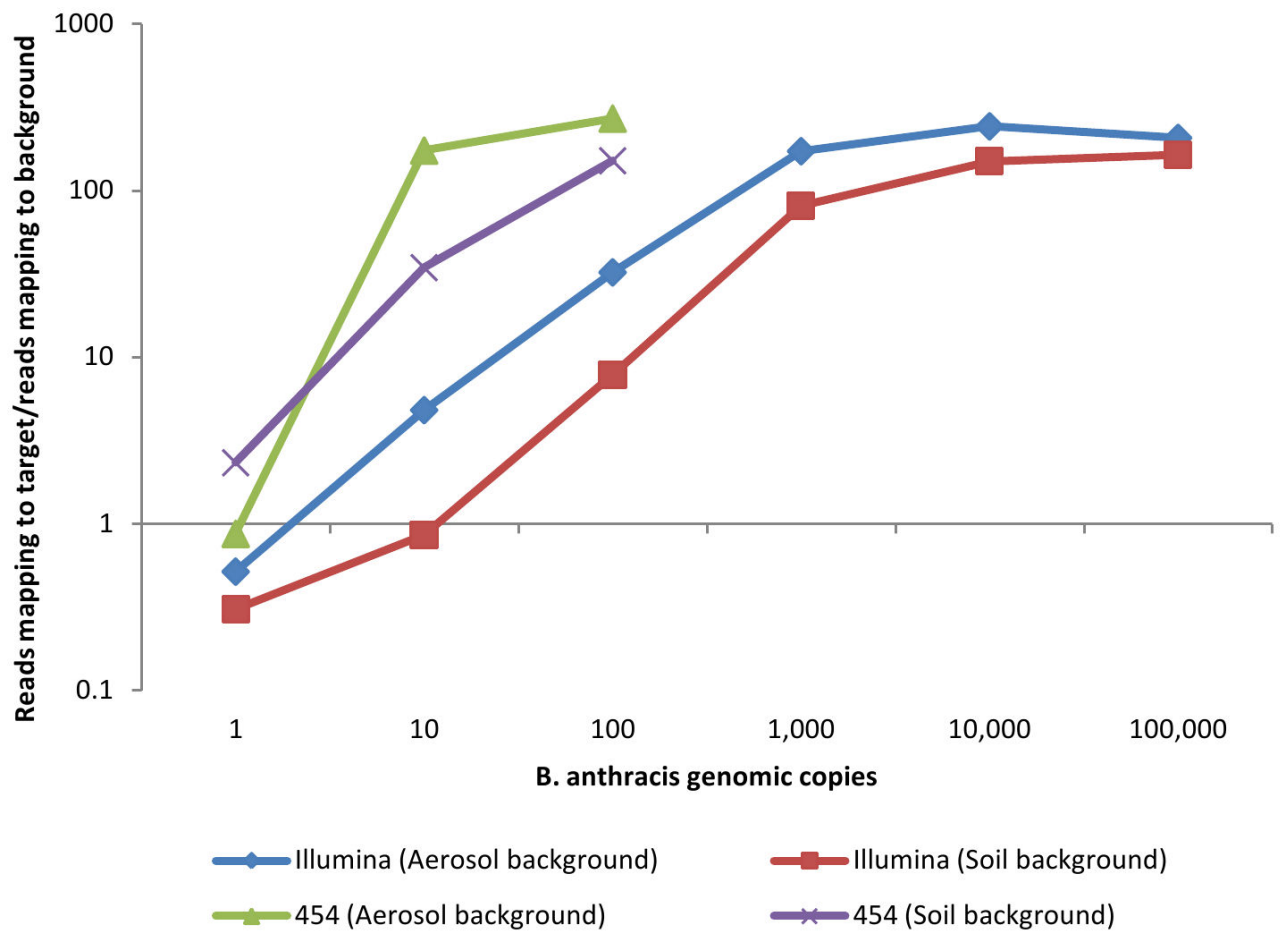
Comparison of *B. anthracis* detection sensitivity via Illumina and 454 sequencing. Sequence reads obtained from environmentally derived DNA spiked with *B. anthracis* DNA were mapped to a target sequence set (*B. anthracis*) or a background set of sequences. Ratios shown were calculated by dividing the number of reads mapped to target reference genomes by the number of reads mapping to background reference genomes.

This observation of slightly higher sensitivity in 454 relative to Illumina sequencing in the soil background (10 versus 100 genome copies/ng soil DNA) could be due to the fact that the 454 analysis produced a higher ratio of reads mapping to target genomes/reads mapping to background genomes (Figure 2). Those reads mapping to target genomes represent detected “signal,” while those reads mapping to our subset of background genomes represent “noise” from which the signal must be distinguished for effective discrimination of *B. anthracis* sequence reads. Our observations are likely due to the increased length of 454 reads, which reduce the likelihood of erroneous, non-specific alignment of sequence reads to background genomes. Superior target/background ratios were also observed with sequence data from aerosol backgrounds compared to soil backgrounds, although this is likely due to the reduced quantity of background DNA used in aerosol relative to soil samples (100 pg compared to 1 ng). Overall, the combined data from Illumina and 454 sequencing indicate accurate detection of *B. anthracis* by analysis of next-generation sequence data when as few as 10-100 genomic copies are present in 1 ng DNA from an environmental sample background.

### Specificity of *B. anthracis* Ames-detection via next-generation sequencing

Following the detection analysis above, studies to determine the specificity of sequencing detection were conducted. The percentage of sequence reads mapping to the *B. anthracis* Ames genome and the pXO1 and pXO2 plasmids was determined and compared to the percentage of reads mapping to two close relatives: *B. thuringiensis* Al Hakam, including the pALH1 plasmid, and *B. cereus* biovar *anthracis* strain CI, including the pCI-X01, pCI-X02, and pBAslCI14 plasmids (Table 2).

**Figure 3:**
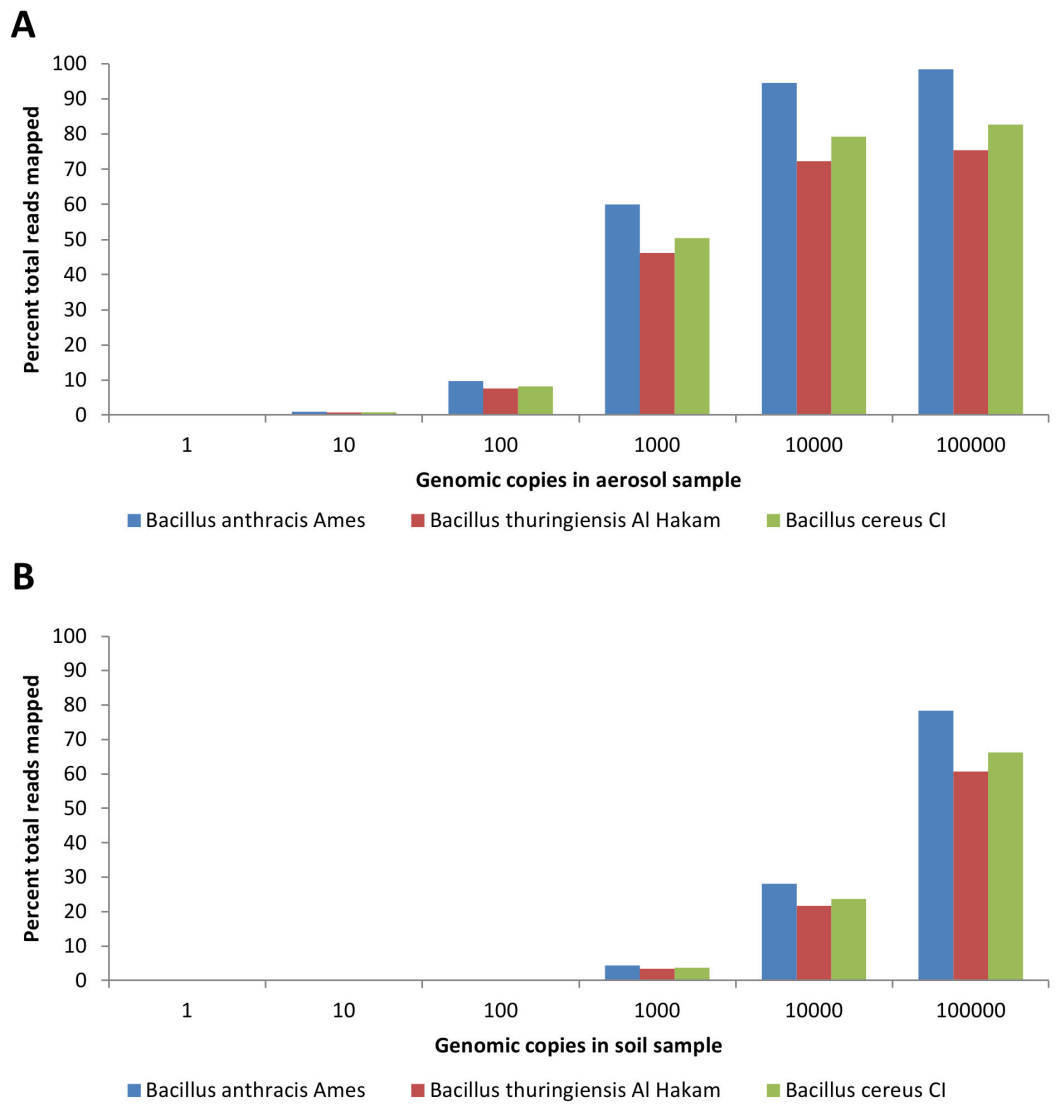
Mapping of Illumina reads to closely related *Bacillus* species. Following sequencing of *B. anthracis*-spiked environmental samples, mapping specificity was examined by determining the percent of total Illumina reads mapping to the closely related species *B. thuringiensis* Al Hakam and *B. cereus* biovar *anthracis* CI. Illumina reads were obtained from A. aerosol background DNA and **B**. soil background DNA samples spiked with increasing amounts of *B. anthracis* DNA.

The proportion of reads mapping to *B. anthracis* Ames was only slightly higher than the proportion of reads mapping to *B. thuringiensis* or *B. cereus* in the soil and aerosol background samples spiked with *B. anthracis* DNA (Figure 3). At the highest spiked-in genome copy number of 100,000, a 1.3-fold increase in the proportion of reads mapping to *B. anthracis* relative to *B. thuringiensis*, and a 1.2-fold increase relative to *B. cereus* was observed. Nearly identical ratios were observed for soil samples spiked with *B. anthracis* DNA. Such narrow distinctions are problematic when aiming for specific detection of *B. anthracis*. The ability to discriminate between strains was thus limited using the approach of comparing all mapped reads due to a high degree of sequence similarity (97.2%) between *B. anthracis* and these two close relatives.

**Figure 4:**
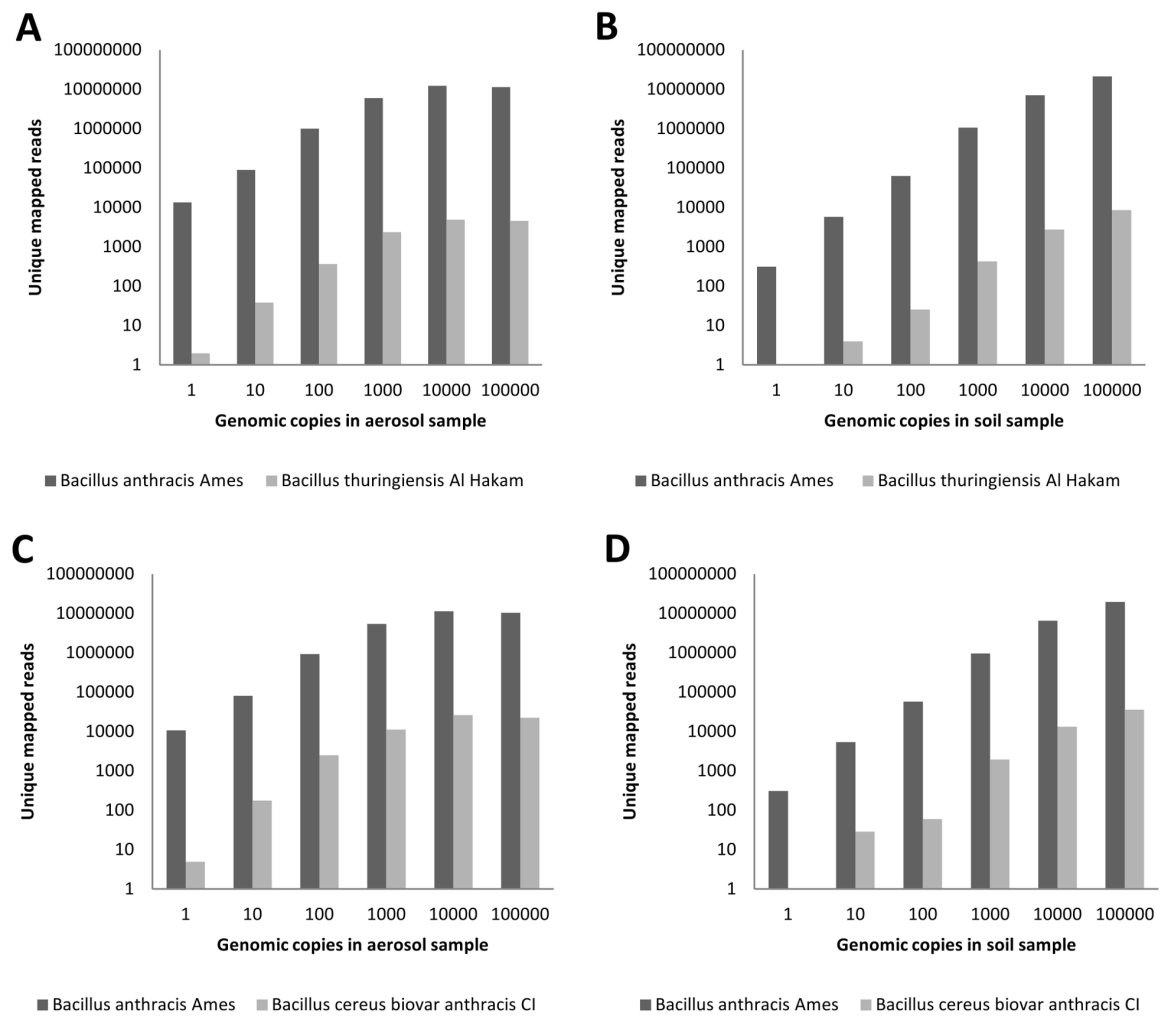
Alignment of uniquely mapped Illumina reads to genomes from *B. anthracis* and two closely-related species. Due to the high degree of sequence similarity among the three examined *Bacillus* species, a unique mapping approach was used. DNA sequencing reads mapping to only *B. anthracis* or one of the two near neighbor species were identified; reads mapping to multiple reference genomes were ignored. This approach facilitated distinction among the three closely related species. The number of uniquely mapped reads for *B. anthracis* is given compared to **A**. *B. thuringiensis* in aerosol samples, **B**. *B. thuringiensis* in soil samples, **C**. *B. cereus* in aerosol samples, and **D**. *B. cereus* in soil samples. This analysis was performed separately for each species – unique reads between *B. anthracis* and *B. thuringiensis* were identified, followed by identification of unique reads between *B. anthracis* and *B. cereus* – thus the results of each comparison are shown in separate charts.

A modified approach was therefore employed, in which only reads mapping to one of the organisms (*B. anthracis* compared to *B. thuringiensis* or *B. cereus*) were considered. Reads that mapped to both or neither reference genome were not considered. The number of uniquely mapped Illumina reads obtained for each *Bacillus* species with increasing copy number is plotted in Figure 4. We observed a greater than 1000-fold increase in the number of reads uniquely mapping to *B. anthracis* compared to *B. thuringiensis* (Figure 4A-B) and a greater than 300-fold increase when compared to *B. cereus* for each sample, when 100 or more *B. anthracis* genome equivalents were present (Figure 4C-D). A modest dose–response effect was observed in number of reads mapping to the two near neighbors, likely due to errant mapping of *B. anthracis*-derived reads to the other very closely related *Bacillus* species. It should be noted that this highly effective differentiation, even at one genome copy, is due to the fact that only the reads uniquely mapping to an organism were considered. By comparison, identification of *B. anthracis* sequence data was less sensitive when all reads were considered for analysis. These data demonstrate that this unique mapping approach can be applied to effectively differentiate *B. anthracis*-specific sequences from closely related *Bacillus* species, even when as few as one genomic equivalent is present.

**Figure 5:**
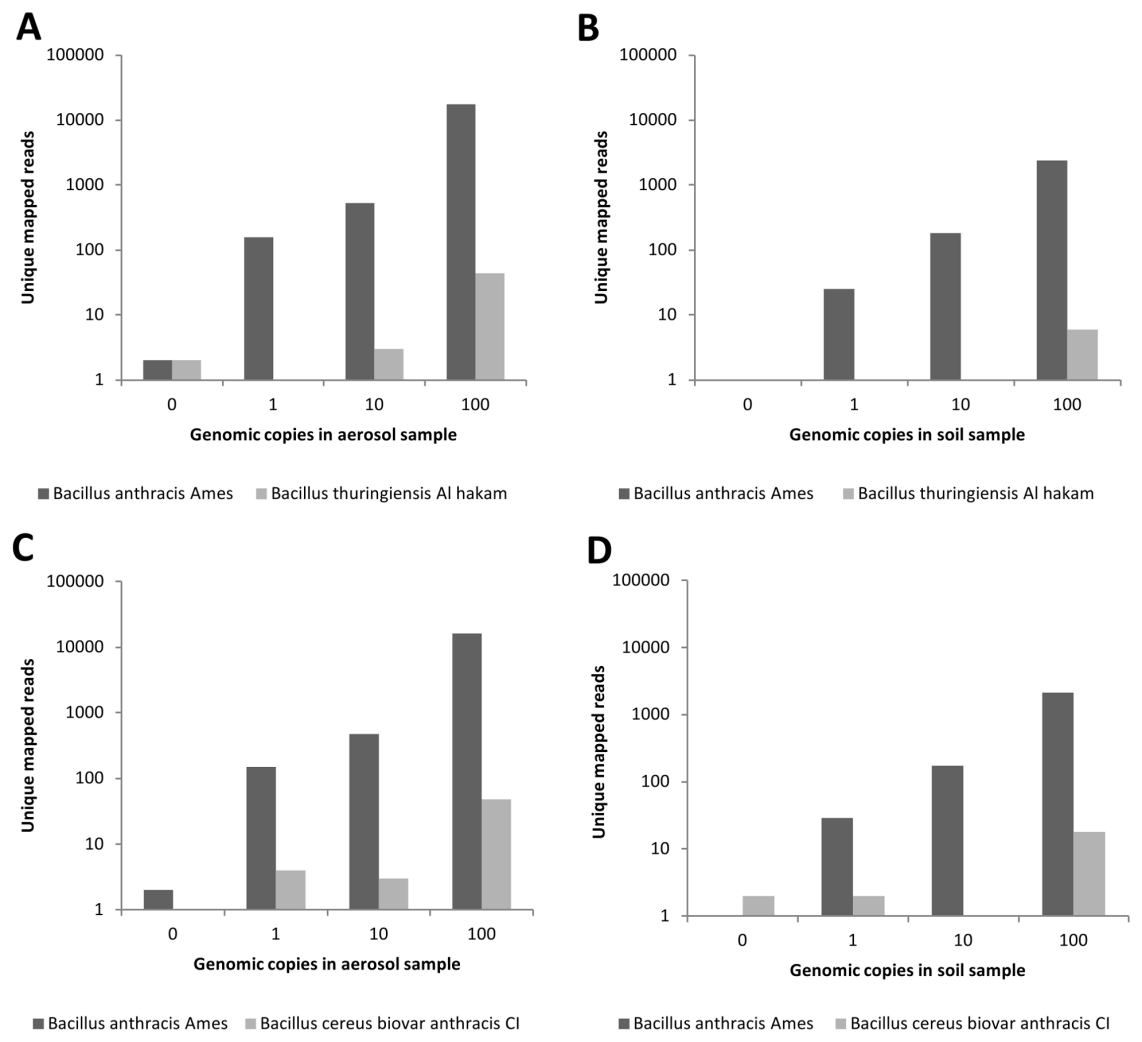
Alignment of unique 454 reads to *B. anthracis* and near-neighbor species. 454 reads mapping only to *B. anthracis* or a close relative, discounting reads mapping to multiple reference genomes, were identified. The number of sequencing reads mapping uniquely to *B. anthracis* Ames DNA are shown compared to **A**. *B. thuringiensis* in aerosol samples, **B**. *B. thuringiensis* in soil samples, **C**. *B. cereus* in aerosol samples, and **D**. *B. cereus* in soil samples. As in Figure 4, this analysis was performed separately for each set of species.

We also applied the above unique read mapping approach to 454 sequence data, with the expectation that 454 reads would map with a greater degree of specificity due to longer read length. 454 sequence reads were mapped to *B. anthracis* Ames genome and plasmids or to the closely related *B. thuringiensis* and *B. cereus* strains and their respective plasmids. Once again, only reads that mapped uniquely to one of the three strains were considered. Results were similar to those obtained by Illumina sequence data analysis, with a higher proportion of reads mapping to *B. anthracis* Ames than to its two near neighbors (Figure 5). The number of reads uniquely mapping to *B. anthracis* exceeded those mapping to *B. thuringiensis* by greater than 300-fold and to *B. cereus* by greater than 100-fold when DNA from either aerosol or soil background samples containing 100 *B. anthracis* genome equivalents was sequenced and the sequences were analyzed. The observed fold differences were less dramatic than those observed with Illumina sequencing above, possibly due to the lower overall quantity of mapped 454 reads, which may have reduced the dynamic range of discrimination. These results confirm our previous observation that the unique mapping analysis approach is a robust method for distinguishing between very closely related *Bacillus* species in complex environmental samples. A potential limitation of this approach is that *B. anthracis* detection is likely to be challenging in samples rich in closely related organisms, such as agricultural samples containing large volumes of *B. thuringiensis*.

### GenBank bowtie analysis of Illumina short read sequence data

We have demonstrated that *B. anthracis* can be specifically identified when sequence data are aligned to a defined set of reference genomes. In order to determine whether identification could be made when considering all available bacterial genomes, a more comprehensive strategy was tested by mapping Illumina sequence data to the full NCBI GenBank database, creating a taxonomic distribution of all organisms in each sample. A local version of the database was used, and only GenBank sequences classified as bacteria or archaea were considered; eukaryotic organisms were not included in this analysis. Mapping was performed using publicly-available bowtie software. This approach was applied to the previously generated shotgun metagenomic Illumina sequence data, as opposed to targeted 16S amplicons, to once again increase resolution among very closely related *Bacillus* species. The shotgun-based approach also provides the potential flexibility of using sequence data to examine the presence of non-bacterial species such as eukaryotic parasites or DNA viruses, should it be deemed necessary.

Taxonomic IDs were sorted according to the number of reads that uniquely mapped to a species, and the number of occurrences of each organism on the basis of total hits was tallied. The 15 taxa with the most hits in each sample were identified, and union sets for all top aerosol and soil hits were created (22 unique species in aerosol and 24 unique species in soil samples) (Table S1). No additional normalization was necessary due to filtering steps that accounted for major sources of expected bias, such as the potential presence of multiple substrain reference genomes. In the aerosol background, *B. anthracis* was identified as the number one (most common) hit by uniquely mapped reads when 1,000 or more genomic copies were present in 100 pg aerosol DNA. When 100, 10, or 1 copies were present, *B. anthracis* represented the 2^nd^, 9^th^, and 67^th^ hit respectively (Table S2). In the soil background, 10,000 or more genomic copies were required in the presence of 1 ng soil DNA for *B. anthracis* to be detected as the number one hit (equivalent to above aerosol results per ng). When 1,000, 100, 10, or 1 copies were present, *B. anthracis* was identified as the 2^nd^, 7^th^, 44^th^, and 293^rd^ hit respectively (Table S3). It should be noted that successful detection does not necessarily demand that *B. anthracis* be detected as the top hit in these analyses. Indeed we would not expect this to be the case in light of the numerous other bacteria found ubiquitously in the environment. Other organisms commonly observed in aerosol backgrounds included *Ralstonia pickettii*, *Cupriavidus* *metallidurans* , and *Delftia* *acidovorans* . Soil samples contained *R. pickettii* and *C* *. metallidurans* , in addition to *Nitrosospira multiformis*. Based on these data, it was confirmed that *B. anthracis* can be identified in an environmental sample when uniquely mapping Illumina short sequence reads to all available bacterial genomes.

A final analysis of the Illumina sequence data was performed by extending the background database to include all GenBank sequences possessing a taxonomic classification (current to April 2013), enabling a more direct comparison with the 454 sequence data analysis below. Examination of all GenBank sequences, including eukaryotes, and keeping all hits with zero mismatches, did not significantly change the outcome with regard to detection of *B. anthracis*. A range of plant and invertebrate organisms were identified in both the soil and aerosol samples (Table S4). When mapping to all organisms and ranking *B. anthracis* on the basis of the total number of reads aligned, *B. anthracis* was among the top 10 ranked organisms with 100 or more genomic copies present in aerosol or soil samples (Table S5).

### MegaBlast analysis of 454 sequence data

We further wanted to demonstrate that the above approach, identifying *B. anthracis* sequence reads when mapping to a comprehensive database, could be applied to longer reads in 454 sequence data A parallel analysis of 454 sequence data was therefore performed using MegaBlast analysis of the NCBI nt database. In this analysis, sequence reads were not required to map uniquely to a species, as longer 454 reads reduce the probability of non-specific alignment. The 15 taxa with the highest numbers of mapped reads in each sample were compiled to create a union set of top hits (27 unique species in aerosol and 32 unique species in soil samples) (Table S6). Due to broad variation in the number of sequencing reads generated for each sample, the occurrences of sequences for each organism were normalized to the total number of reads in the sample. These results show approximate successive 10-fold increases in the number of sequence reads mapped to *B. anthracis* with each 10-fold increase in *B. anthracis* genomic copy number. Due to use of the fully comprehensive reference database, sequence reads were also mapped to mammalian, fungal, and plant species. These data demonstrate that sequence reads are effectively mapped to *B. anthracis* in an environmental sample using 454 sequence data, even when mapping reads to all available NCBI reference sequences.

### Microarray-based detection of *B. anthracis* in aerosol and soil samples

The detection sensitivity of a microbial census microarray, developed at Lawrence Livermore National Laboratory (LLNL), was tested to determine its applicability for detection of biothreat agents from environmental samples. This array contained two distinct probe categories: census and detection. Detection probes were designed to be conserved across multiple sequences from within a family, but not across families or kingdoms. Such probes aim to detect known organisms or discover novel organisms exhibiting some homology to species whose genomes have previously been sequenced, particularly in those regions known to be conserved. The Lawrence Livermore Microbial Detection Array (LLMDA) was previously designed using this approach [20]. In contrast, census probes represent the least conserved regions, and are the most strain or isolate-specific probes (Table S7). Such census probes should provide higher level resolution among known species and strains to facilitate forensic discrimination.

The census array was tested using the same serially-diluted *B. anthracis* Ames genomic DNA spiked into either aerosol or soil samples, followed by whole genome amplification. DNA from each sample was fluorescently labeled and hybridized to the census array in duplicate experiments. A statistical method was developed to analyze the array data, by estimating the log likelihood of the observed probe intensities as a function of the combination of targets present in the sample, and performing greedy maximization to find a locally optimal set of targets.

In the presence of 100 pg aerosol background DNA, 100 genome equivalents of *B. anthracis* DNA were required for successful microarray detection of these sequences in both replicates (Table 3), while 10 genome copies were detected in one replicate. In the presence of 1 ng soil background DNA, 1000 genome equivalents were required for detection in both replicates, while 100 genome copies were detected in one replicate. The organisms with the highest detection likelihood scores in each sample are shown in Table S8. When normalized to amount of background DNA present, these results demonstrate microarray-based detection of 1000 bacilli in the presence of one nanogram of environmental background DNA. A summary of the detection thresholds and characteristics of each method employed in this study is given in Table 4.

**Table 3:** Detection of *B. anthracis* DNA in environmental background by census microarray.

***Aerosolbackground***						
***B. anthracisgenome* copy #**	100,000	10,000	1,000	100	10	1
**Array detection**	*B. anthracis*	*B. anthracis*	*B. anthracis*	*B. anthracis*	*B. anthracis* *	N/D
***Soilbackground***						
***B. anthracisgenome* copy #**	100,000	10,000	1,000	100	10	1
**Array detection**	*B. anthracis*	*B. anthracis*	*B. anthracis*	*B. anthracis* *	N/D	N/D

* Detection of *B. anthracis* by only one array replicate. The second replicate yielded detection of *Bacillus* sp. in aerosol background and *B. cereus* in soil background. N/D:B*. anthracis* not detected. Detection of *B. anthracis* DNA was examined in the context of 100 pg aerosol DNA or 1 ng soil DNA.

**Table 4:** Summary of *B. anthracis* detection in environmental samples via genomic technologies.

Assay	Minimum genome copies required for detection per ng environmental DNA		Assay properties			
	**Aerosol background**	**Soil background**	**Resolution**	**Computational time**	**Throughput**	**Cost**
*Illumina sequencing (BWA)*	100	100	++++	++++	++	+++
*454 sequencing (gsMapper)*	100	10	++++	++++	++	+++
*Specific detection (unique read mapping approach)*	1	1	++++	++++	++	+++
*Microarray*	1000*	1000*	++	+	+++	++

* For microarray, detection occurs at 100 copies when using the *Bacillus* genus as a threshold, instead of the *B. anthracis* species.

It was noted that a number of the positively identified species were either geographically foreign to the collection site or were not expected to be observed in conventional soil or aerosol environments due to their affinity for saline, anoxic, or low temperature conditions. Among these organisms were *Tolumonas* *auensis* *,* *Thioalkalivibrio* *, Psychroflexus torquis, Haliangium ochraceum*, and *Photobacterium profundum*. To further evaluate the potential presence of these bacteria, the array probes that were identified as positive hits above the 0.95 quantile of negative controls were aligned via BLAST to the Illumina metagenomic sequence data from the soil samples collected in this study. The number of array probes with positive matches to the metagenome and the number of unique reads with matches to these probes are given in Table S9. With the exception of *H. ochraceum*, a substantial number of probes aligned to sequence data from the soil metagenomes, in some cases corresponding to several thousand unique reads. These data could point toward the presence of these organisms in the soil samples interrogated, or alternatively, these species may exhibit substantial homology with organisms in these samples that have not yet been sequenced and archived. As mentioned above, relatively few positive probes corresponding to *H. ochraceum* aligned to metagenomic sequence reads. Probes designed for this particular organism contained a relatively high average GC content of 68.4%, which may have conferred enhanced promiscuity in these probes, and could explain the elevated observance of *H. ochraceum* via the array technology.

## Discussion

The life cycle and virulence of *B. anthracis* distinguish it as an organism with a high potential for use as a bioterrorism agent, as well as being problematic as a natural human and animal pathogen in many parts of the world. Efforts toward detection of *B. anthracis* are fraught with multiple difficulties, including the presence of extraneous contaminants, low bacterial numbers, and non-cultivable bacilli within suspect samples [21]. Studies using a variety of methods for detection of *B. anthracis* have been performed in the past, and have been effectively summarized in several reviews [8,11,21]. Methods applied include RT-PCR, microarray, ELISA-based immunoassays, spectroscopy, mass spectrometry, biosensor assays, and Sanger sequencing. This study demonstrates that analysis of next-generation sequencing data and detection microarrays represent sensitive and specific complementary techniques for *B. anthracis* identification.

The increasing availability and falling cost of next-generation sequencing technology provide an opportunity to perform whole genome analyses of pathogenic microbes, providing a breadth of information not available from more limited and focused genetic protocols such as PCR or Sanger sequencing. Next-generation sequencing has been used to characterize isolates of *B. anthracis* and successfully identified SNPs corresponding to distinct strains [22]. The Amerithrax investigation of the 2001 anthrax letters used whole genome sequencing and comparative analyses to identify unique genomic characteristics of the *B. anthracis* strain sent in the letters, validating the forensic potential of this technology [8,23]. These and other similar efforts have used purified DNA from isolated *Bacillus* strains [24,25], identifying unique and minute genetic characteristics [25]. Analysis of next-generation sequencing data for detection of biothreat organisms has not, however, been validated in the context of an environmental background [8]. Since these are the sample types most likely to be encountered in the event of bacillary release, screening for *B. anthracis* genomic DNA in aerosol and soil backgrounds was performed after addition of titrated genomic equivalents to determine detection efficacy. A whole genome approach was employed to increase species resolution and provide comprehensive breadth of organism detection.

### Approaches for identification of *B. anthracis* via analysis of next-generation sequence data

Samples in this study were subjected to whole genome amplification (WGA) and evaluated on two different sequencing platforms: Illumina and 454. WGA does have the potential to alter relative representation of microbial communities in the resultant amplicons due to amplification bias, depending on the methods applied. For microbial detection purposes, however, WGA is an important step for maximizing the detection of very low bacillary genomic copy numbers, even if these sequences have a negative bias and are amplified less frequently. Additionally, the Phi29-based REPLI-g system employed in this study has been demonstrated to show high uniformity and minimal bias in both detection of microbial genes and genotyping, thereby minimizing potential effects on resultant sequence data [26,27].

Sequencing data from each sample were analyzed using two different procedures. The first method used Burrows-Wheeler Aligner (Illumina reads) and gsMapper (454 reads) software to map reads to specific reference genomes. This approach demonstrated increasing numbers of reads mapped to *B. anthracis* target genomes without a corresponding increase in reads mapped to background reference genomes. The set of background genomes was selected to examine mapping sensitivity and observe sample variation; however, it should be noted that selection of a different set of reference genomes could alter the observed detection sensitivity. This issue was examined in the latter experiments, in which reads were mapped to the complete GenBank database.

We did observe, in using the above approach, that the number of sequence reads mapping to target reference genomes was generally higher than expected, given the relative proportion of *B. anthracis* DNA present. This discrepancy could be due to higher overall quality of spiked *B. anthracis* DNA relative to environmental background bacterial DNA, which might have led to some amplification bias. The second method used bowtie (Illumina reads) and MegaBlast (454 reads) to query the full NCBI GenBank database for alignment. The mapping approach using a defined number of reference genomes was much faster, with run times of several minutes compared to hours/days for the more comprehensive NCBI queries. Aligning to the full GenBank reference database will, however, provide a more comprehensive approximation of all known organisms in a given sample. Both methods gave similar results with regard to the number of reads aligned to *B. anthracis* relative to the amount of *B. anthracis* genomic equivalents present.

The full GenBank analysis revealed a number of additional species present in environmental samples, particularly those belonging to the genera *Ralstonia* and *Cupriavidus* . These species are adapted to survival in terrestrial, soil environments, and are not known to be pathogenic. They represent microorganisms likely to be observed as background when comparable analyses of environmental samples are performed. It is therefore important to observe that their presence does not adversely affect our ability to detect *B. anthracis*. While the distinct sequencing protocols were all shown to detect the presence of *B. anthracis*, they did, in some cases, differ with regard to assessment of the predominant background taxa. These differences can in part be attributed to the different methodologies applied, as differing read lengths may impact degree of mapping efficiency. Observed discrepancies should be viewed meaningfully when interpreting results obtained from shotgun metagenomic data analysis. Further examination on broader detection of microbial populations using different shotgun metagenomic sequencing protocols is an area of active interest and will be the subject of future studies.

### Distinguishing *B. anthracis* from near genetic neighbors

One of the major challenges relating to specific detection of *B. anthracis* is the similarity of its genome to very closely related *B. cereus* and *B. thuringiensis* strains. In fact, some sequence analyses have indicated *B. anthracis* could be classified as a lineage of *B. cereus* [28]. While the majority of *B. cereus* and *B. thuringiensis* strains are not pathogenic in humans and other mammals, a small subset, often genetically very closely linked to *B. anthracis*, are highly pathogenic opportunistic pathogens [29,30]. These are the strains with genomes containing sequences most similar to those found in *B. anthracis* [29,31,32]. It has been suggested that the uniqueness of *B. anthracis* is actually a more complex product of co-evolution of the genome with its corresponding plasmids [11,31]. Similarly, within *B. anthracis*, individual strains share a tremendous degree of similarity, leading to suggestions that *B. anthracis* represents the most genetically homogenous known bacterial species [11]. All of these issues present challenges to the specific identification of this pathogen in complex mixtures using nucleic acid-based detection methods.

The comprehensive nature of sequencing was expected to provide heightened specificity in making these difficult distinctions. Indeed, using our unique analysis approach, a greater than 300-fold increase was observed in the number of reads mapping uniquely to *B. anthracis*, compared to the number of reads mapping to its near neighbors *B. cereus* biovar *anthracis* str. CI and *B. thuringiensis* str. Al Hakam. Although both sequencing technologies were capable of making these distinctions, greater differentiation power was observed when examining Illumina reads versus 454 reads, based on the ratio of uniquely mapped reads between *B. anthracis* and close relatives. Taken together, these results suggest that the analysis of next-generation sequencing data with these unique mapping strategies could be a highly effective way to approach the problematic task of distinguishing *B. anthracis* from other members of the *B. cereus* group.

These data analyses provide broader insight into interference in mapping classification caused by sequence similarity among bacterial genomes, particularly when mapping short reads in closely-related but distinct bacterial species. The use of reads that uniquely map to a given species is an important step toward remedying the detection of false-positives during microbiome characterization. One potential future difficulty in this approach lies in the fact that, without prior information, it may be difficult to estimate the exact quantity of *B. anthracis* in environmental samples via analysis of sequence data. This would be a function of the proportion of total reads mapping to the target database, and the size, quantity, and diversity of a microbial background will never be identical between distinct samples. One possible solution for this problem could include the use of fixed standard samples for development of a geometric distribution model for predicting the amount of target organism present in a mixed sample. Such models are beyond the scope of this study, but will be the subject of future efforts.

There is an important limitation to the above approach, namely the sensitivity and specificity of *B. anthracis* detection in samples rich in closely related species, such as agricultural backgrounds rich in *B. thuringiensis* and *B. cereus*. Detection of *B. anthracis* in such samples, particularly if it is present in trace amounts, is expected to be problematic, and may result in false-negative results. We have shown that this approach is capable of distinguishing between the target organism and its near-neighbors, with the signal for *B. anthracis* being several orders of magnitude greater than the signal for its closest relatives. This signal is present even in the samples with the lowest copy numbers of spiked-in *B. anthracis*. However, signal for both *B. cereus* and *B. thuringiensis* were still observable in our samples. While it is unknown whether these species were present in the soil and aerosol samples employed in this study, the number of reads mapping to these genomes is directly proportional to the number of reads mapped to the spiked-in *B. anthracis*. Thus we suspect that these represent false positive signals related to the presence of *B. anthracis* DNA. It is expected that the presence of closely related organisms would complicate the detection of a target organism. Detection of trace amounts of *B. anthracis*, or estimation of its frequency in backgrounds rich in close relatives is therefore a challenging problem, regardless of whether whole shotgun sequencing or other approaches are used, and will be a subject of future work.

### Microarray technology as a complementary detection method

This study has demonstrated that next-generation sequencing is specific, sensitive, high resolution, and has the potential to serve as a critical component of the biosurveillance armamentarium. A comprehensive microbial census array was also applied in this study to assess a complementary broad spectrum and cost-effective detection technology. Two classes of probes were included on the array, census and detection, to maximize the capacity for identification of sequenced and novel microbes and to facilitate high confidence at multiple taxonomic levels. The inclusion of strain-specific census probes contributed unique resolution to the detection capacity of this array. A number of the organisms detected by the census array differed from the top hits observed in the bowtie and MegaBlast analyses. Given the distinct approaches, however – analysis of microarray hybridization versus sequence data mapping – we would expect this to be the case. Moreover, the array probes do in some cases represent species outside of the NCBI reference sequences used for mapping. The array results also yielded detection of bacterial species not anticipated in conventional environmental samples such as the halophiles *Haliangium ochraceum,* *Thioalkalivibrio* , and *Photobacterium profundum*, and the psychrophile *Psychroflexus torquis*. Subsequent analysis of the probe sequences corresponding to these species did yield alignment to metagenomic sequence data obtained from the same samples, but also revealed instances of probes which may demonstrate heightened promiscuity. All of the above observations point toward the utility of validation via orthogonal methods to corroborate the detection of atypical microorganisms.

With detection of 1000 *B. anthracis* genome equivalents/ng background DNA, the observed sensitivity was one order of magnitude lower than that of the next-generation sequencing approach. However, if we include the detection of the *Bacillus* genus instead of only the *B. anthracis* species, detection is observed at 100 genome equivalents/ng background DNA. At 100 copies in the soil background for instance, one of two replicates detected *B. anthracis*, with the alternate replicate detecting *B. cereus*. Although it demonstrated lower sensitivity than next-generation sequencing, the census array provides a platform that is, at present, more cost-effective than sequencing, while maintaining parallel detection of many organisms in one sample. Moreover, the array does not require extensive bioinformatics analyses of sequence data with a multi-CPU computer cluster, and can be packaged with a pre-designed array analysis platform. This platform may be run at minimal time and cost by an individual without prior bioinformatics training, or the requirement for time- and computation-intensive comparisons to large genomic reference databases. These features support the array as a complementary screening technology for biothreat agent detection, whereupon subsequent sequencing may be performed if it is determined that heightened genomic resolution is desirable.

Finally, it is important to note that analysis of next-generation sequencing data has open potential for bringing to light new variants of *B. anthracis*. The combined use of sequencing and high-density detection microarrays allows for highly comprehensive detection of potential biothreats, providing detailed genomic information useful for diagnostic and forensic purposes. Increasing availability of sequencing technology and its amplified presence in laboratories worldwide should make next-generation sequencing an important component of the biodefense surveillance strategy moving forward, particularly in cases where anomalous and emergent strains are a concern.

## Supporting Information

Figure S1 **Absolute numbers of sequencing reads mapping to specified reference genomes.** Increasing genome equivalents of *B. anthracis* DNA were spiked into environmental background nucleic acid and subjected to whole genome amplification and next-generation sequencing. Resultant reads were mapped to either a target set (*B. anthracis*) or a background set of sequences. Shown are total reads mapped to the target and background reference groups (logarithmic scale) for A. Illumina reads from the aerosol background, B. Illumina reads from the soil background, C. 454 reads from the aerosol background, and D. 454 reads from the soil background.
(TIF)

Table S1 **Mapping of Illumina sequence reads to the GenBank reference database.** The top 15 taxonomic IDs observed in Illumina sequence data from each *B. anthracis*-spiked environmental sample were compiled into a union set of prominently observed species. Each species is listed with its corresponding number of total mapped reads. Species identified by Illumina sequencing in both aerosol and soil samples are shown.
(DOCX)

Table S2 **Top 15 species detected in aerosol samples spiked with *B. anthracis*.** Species are sorted by number of Illumina reads mapped by bowtie to only one bacterial species (*B. anthracis* hits in bold).
(DOCX)

Table S3 **Top 15 species detected in soil samples spiked with *B. anthracis*.** Species are sorted by number of Illumina reads mapped by bowtie to only one bacterial species (*B. anthracis* hits in bold).
(DOCX)

Table S4 **The top 15 non-bacterial organisms identified at the lowest *B. anthracis* genome copy spike-in level in aerosol and soil samples.**
(DOCX)

Table S5 **Detection ranking of *B. anthracis*, as determined by total number of mapped reads, following mapping (zero mismatches) of sequence data to all organisms versus bacteria only.**
(DOCX)

Table S6 **Mapping of 454 sequencing reads to the GenBank reference database.** The top 15 taxonomic IDs observed in 454 sequence data from each *B. anthracis*-spiked environmental sample were compiled into a union set of species most prominently observed. Each species is listed with its corresponding normalized number of mapped reads (normalized to total reads for each given sample). The species identified by 454 sequencing in both aerosol and soil samples are shown.
(DOCX)

Table S7 **Summary of microbial sequences represented on the census array.**
(DOCX)

Table S8 **Top hit organisms detected by the census array in *B. anthracis*-spiked aerosol and soil samples.**
(DOCX)

Table S9 **Census array probe sequence matches to metagenomic sequence data from soil samples for bacterial species not anticipated in conventional environmental samples.**
(DOCX)
