# Supplementary figures and images for: Detection of Bacillus anthracis DNA in Complex Soil and Air Samples Using Next-Generation Sequencing
Source: PLoS One. 2013 Sep 9;8(9):e73455. doi: 10.1371/journal.pone.0073455 (PMC3767809; doi:10.1371/journal.pone.0073455)

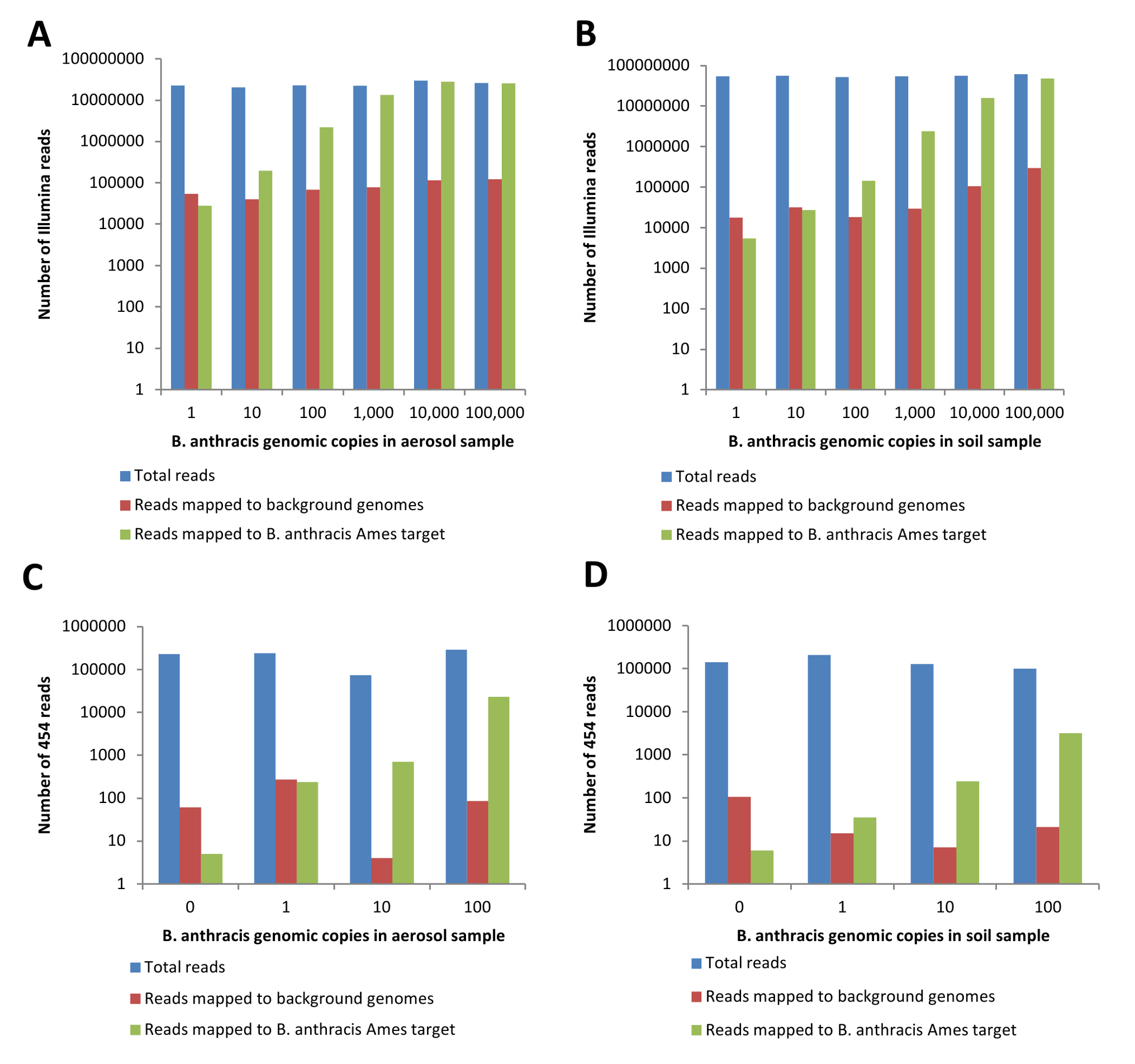

Supplement: Figure S1 — Absolute numbers of sequencing reads mapping to specified reference genomes. Increasing genome equivalents of B. anthracis DNA were spiked into environmental background nucleic acid and subjected to whole genome amplification and next-generation sequencing. Resultant reads were mapped to either a target set (B. anthracis) or a background set of sequences. Shown are total reads mapped to the target and background reference groups (logarithmic scale) for A. Illumina reads from the aerosol background, B. Illumina reads from the soil background, C. 454 reads from the aerosol background, and D. 454 reads from the soil background. (TIF) [file pone.0073455.s001.tif]
