# Supplementary material for: Detection of Bacillus anthracis DNA in Complex Soil and Air Samples Using Next-Generation Sequencing
Source: PLoS One. 2013 Sep 9;8(9):e73455. doi: 10.1371/journal.pone.0073455 (PMC3767809; doi:10.1371/journal.pone.0073455)
Supplement: Table S1 — Mapping of Illumina sequence reads to the GenBank reference database. The top 15 taxonomic IDs observed in Illumina sequence data from each B. anthracis-spiked environmental sample were compiled into a union set of prominently observed species. Each species is listed with its corresponding number of total mapped reads. Species identified by Illumina sequencing in both aerosol and soil samples are shown. (DOCX) [file pone.0073455.s002.docx]

**Table S1. Mapping of Illumina sequence reads to the GenBank reference database.** The top 15 taxonomic IDs observed in Illumina sequence data from each *B. anthracis*-spiked environmental sample were compiled into a union set of prominently observed species. Each species is listed with its corresponding number of total mapped reads. Species identified by Illumina sequencing in both aerosol and soil samples are shown.

|  | ***B. anthracis* genome equivalents** | | | | | |
| --- | --- | --- | --- | --- | --- | --- |
| **Organism** | **1** | **10** | **100** | **1000** | **10000** | **100000** |
| ***Aerosol sample mapped reads*** | | | | | | |
| *Acidovorax* sp. JS42 | 29228 | 28766 | 32643 | 9527 | 3232 | 217 |
| *Bacillus anthracis* | 26484 | 194577 | 2218377 | 13397458 | 28114696 | 25585167 |
| *Bacillus atrophaeus* | 126 | 450 | 9581 | 35912 | 76225 | 78358 |
| *Bacillus cereus* | 26404 | 187905 | 2152273 | 12970946 | 27207534 | 24755451 |
| *Bacillus thuringiensis* | 23591 | 169908 | 1960100 | 11710402 | 24439877 | 22217509 |
| *Bacillus weihenstephanensis* | 7301 | 50502 | 593804 | 3497920 | 7258565 | 6729154 |
| *Bradyrhizobium japonicum* | 49743 | 24591 | 39442 | 18533 | 1741 | 356 |
| *Bradyrhizobium* sp. BTAi1 | 60249 | 22741 | 40357 | 25080 | 1510 | 285 |
| *Bradyrhizobium* sp. ORS278 | 30122 | 14466 | 23341 | 10673 | 1040 | 110 |
| *Cupriavidus metallidurans* | 187166 | 221742 | 166344 | 49516 | 6533 | 877 |
| *Cupriavidus necator* | 104660 | 101795 | 94635 | 31897 | 4343 | 565 |
| *Cupriavidus pinatubonensis* | 92029 | 89082 | 86594 | 25230 | 4096 | 550 |
| *Cupriavidus taiwanensis* | 102033 | 98663 | 93067 | 26933 | 4277 | 617 |
| *Delftia acidovorans* | 57103 | 239451 | 47525 | 20349 | 2762 | 760 |
| *Hyphomicrobium denitrificans* | 12691 | 3665 | 5721 | 6502 | 684 | 34 |
| *Pantoea vagans* | 13333 | 1930 | 3312 | 1613 | 192 | 51 |
| *Propionibacterium acnes* | 239 | 195 | 942 | 2706 | 4932 | 5735 |
| *Pseudomonas aeruginosa* | 29624 | 37812 | 22575 | 11734 | 1703 | 791 |
| *Ralstonia pickettii* | 521846 | 600229 | 442592 | 153781 | 19234 | 2269 |
| *Ralstonia solanacearum* | 190355 | 197290 | 169308 | 57627 | 8634 | 1448 |
| *Rhodopseudomonas palustris* | 45250 | 23305 | 37835 | 16177 | 1542 | 180 |
| *Stenotrophomonas maltophilia* | 8659 | 6955 | 9853 | 3126 | 236 | 35 |
| ***Soil sample mapped reads*** | | | | | | |
| *Acidovorax* sp. JS42 | 9656 | 13308 | 12962 | 8343 | 9542 | 1930 |
| *Arthrobacter* sp. | 1182 | 3205 | 2213 | 821 | 789 | 1655 |
| *Bacillus anthracis* | 4609 | 23068 | 142371 | 2376583 | 15867654 | 48222281 |
| *Bacillus cereus* | 5614 | 23579 | 138205 | 2300101 | 15357112 | 46666754 |
| *Bacillus megaterium* | 2520 | 11563 | 6022 | 12526 | 67539 | 209191 |
| *Bacillus thuringiensis* | 2505 | 19624 | 124049 | 2067500 | 13847326 | 42113778 |
| *Bacillus weihenstephanensis* | 1658 | 11020 | 39495 | 641307 | 4266819 | 13116108 |
| *Bradyrhizobium* sp. BTAi1 | 1481 | 2621 | 2305 | 2400 | 775 | 278 |
| *Cupriavidus metallidurans* | 57078 | 71004 | 64133 | 43764 | 63261 | 10281 |
| *Cupriavidus necator* | 37036 | 45260 | 41907 | 28053 | 38791 | 5783 |
| *Cupriavidus pinatubonensis* | 31026 | 37692 | 34539 | 23864 | 32063 | 5073 |
| *Cupriavidus taiwanensis* | 35364 | 42641 | 39651 | 26711 | 36560 | 5628 |
| *Delftia acidovorans* | 22714 | 40129 | 21583 | 12085 | 15706 | 4802 |
| *Hyphomicrobium denitrificans* | 3073 | 4534 | 3385 | 2953 | 1904 | 389 |
| *Magnetospirillum gryphiswaldense* | 6455 | 7361 | 7118 | 6960 | 4616 | 867 |
| *Nitrosospira multiformis* | 37376 | 44282 | 42772 | 42300 | 31365 | 5684 |
| *Propionibacterium acnes* | 388 | 824 | 300 | 1231 | 7675 | 13526 |
| *Pseudomonas aeruginosa* | 14592 | 19281 | 16919 | 11167 | 12694 | 6480 |
| *Pseudomonas fluorescens* | 3073 | 3713 | 3324 | 2220 | 4150 | 860 |
| *Ralstonia pickettii* | 141350 | 194416 | 182124 | 137964 | 119288 | 20909 |
| *Ralstonia solanacearum* | 64087 | 89217 | 79182 | 60271 | 56994 | 11176 |
| *Rhodococcus erythropolis* | 870 | 1310 | 4778 | 4701 | 4511 | 12397 |
| *Stenotrophomonas maltophilia* | 6051 | 4058 | 3377 | 2400 | 5526 | 537 |
| Uncultured bacterium | 45567 | 64289 | 46504 | 53432 | 113518 | 265551 |
