# Supplementary material for: Detection of Bacillus anthracis DNA in Complex Soil and Air Samples Using Next-Generation Sequencing
Source: PLoS One. 2013 Sep 9;8(9):e73455. doi: 10.1371/journal.pone.0073455 (PMC3767809; doi:10.1371/journal.pone.0073455)
Supplement: Table S2 — Top 15 species detected in aerosol samples spiked with B. anthracis. Species are sorted by number of Illumina reads mapped by bowtie to only one bacterial species (B. anthracis hits in bold). (DOCX) [file pone.0073455.s003.docx]

**Table S2. Top 15 species detected in aerosol samples spiked with *B. anthracis*.** Species are sorted by number of Illumina reads mapped by Bowtie to only one bacterial species (*B. anthracis* hits in bold).

| *B. anthracis* genome equivalents | | | | | |
| --- | --- | --- | --- | --- | --- |
| 1 | **10** | **100** | **1,000** | **10,000** | **100,000** |
| *Ralstonia pickettii* | *Ralstonia pickettii* | *Ralstonia pickettii* | ***Bacillus anthracis*** | ***Bacillus anthracis*** | ***Bacillus anthracis*** |
| *Cupriavidus metallidurans* | *Cupriavidus metallidurans* | ***Bacillus anthracis*** | *Ralstonia pickettii* | *Ralstonia pickettii* | *Ralstonia pickettii* |
| *Ralstonia solanacearum* | *Ralstonia solanacearum* | *Cupriavidus metallidurans* | *Cupriavidus metallidurans* | *Cupriavidus metallidurans* | *Bacillus cereus* |
| *Bradyrhizobium* sp. BTAi1 | *Delftia acidovorans* | *Ralstonia solanacearum* | *Bradyrhizobium* sp. BTAi1 | *Bacillus cereus* | *Delftia acidovorans* |
| *Bradyrhizobium japonicum* | *Cupriavidus necator* | *Bradyrhizobium* sp. BTAi1 | *Ralstonia solanacearum* | *Ralstonia solanacearum* | *Cupriavidus metallidurans* |
| *Delftia acidovorans* | *Bradyrhizobium* sp. BTAi1 | *Bradyrhizobium japonicum* | *Bradyrhizobium japonicum* | *Delftia acidovorans* | *Bacillus thuringiensis* |
| *Rhodopseudomonas palustris* | *Bradyrhizobium japonicum* | *Delftia acidovorans* | *Delftia acidovorans* | *Bradyrhizobium* sp. BTAi1 | *Propionibacterium acnes* |
| *Cupriavidus necator* | *Cupriavidus taiwanensis* | *Rhodopseudomonas palustris* | *Rhodopseudomonas palustris* | *Bradyrhizobium japonicum* | *Ralstonia solanacearum* |
| *Cupriavidus taiwanensis* | ***Bacillus anthracis*** | *Cupriavidus necator* | *Hyphomicrobium denitrificans* | *Hyphomicrobium denitrificans* | *Bradyrhizobium* sp. BTAi1 |
| *Cupriavidus pinatubonensis* | *Cupriavidus pinatubonensis* | *Cupriavidus taiwanensis* | *Cupriavidus necator* | *Rhodopseudomonas palustris* | *Bacillus atrophaeus* |
| *Hyphomicrobium denitrificans* | *Rhodopseudomonas palustris* | *Cupriavidus pinatubonensis* | *Cupriavidus taiwanensis* | *Bacillus thuringiensis* | *Bacillus weihenstephanensis* |
| *Bradyrhizobium* sp. ORS278 | *Pseudomonas aeruginosa* | *Bradyrhizobium* sp. ORS278 | *Bradyrhizobium* sp. ORS278 | *Cupriavidus taiwanensis* | *Bradyrhizobium japonicum* |
| *Pantoea vagans* | *Hyphomicrobium denitrificans* | *Acidovorax* sp. JS42 | *Cupriavidus pinatubonensis* | *Cupriavidus necator* | *Rhodopseudomonas palustris* |
| *Pseudomonas aeruginosa* | *Stenotrophomonas maltophilia* | *Hyphomicrobium denitrificans* | *Stenotrophomonas maltophilia* | *Cupriavidus pinatubonensis* | *Cupriavidus taiwanensis* |
| *Stenotrophomonas maltophilia* | *Bradyrhizobium* sp. ORS278 | *Pantoea vagans* | *Pseudomonas aeruginosa* | *Bacillus weihenstephanensis* | *Cupriavidus necator* |
