# Supplementary material for: Detection of Bacillus anthracis DNA in Complex Soil and Air Samples Using Next-Generation Sequencing
Source: PLoS One. 2013 Sep 9;8(9):e73455. doi: 10.1371/journal.pone.0073455 (PMC3767809; doi:10.1371/journal.pone.0073455)
Supplement: Table S3 — Top 15 species detected in soil samples spiked with B. anthracis. Species are sorted by number of Illumina reads mapped by bowtie to only one bacterial species (B. anthracis hits in bold). (DOCX) [file pone.0073455.s004.docx]

**Table S3. Top 15 species detected in soil samples spiked with *B. anthracis***. Species are sorted by number of Illumina reads mapped by Bowtie to only one bacterial species (*B. anthracis* hits in bold).

| *B. anthracis* genome equivalents | | | | | |
| --- | --- | --- | --- | --- | --- |
| 1 | **10** | **100** | **1,000** | **10,000** | **100,000** |
| *Ralstonia pickettii* | *Ralstonia pickettii* | *Ralstonia pickettii* | *Ralstonia pickettii* | ***Bacillus anthracis*** | ***Bacillus anthracis*** |
| *Nitrosospira multiformis* | *Nitrosospira multiformis* | *Nitrosospira multiformis* | ***Bacillus anthracis*** | *Ralstonia pickettii* | *Ralstonia pickettii* |
| *Cupriavidus metallidurans* | *Cupriavidus metallidurans* | *Cupriavidus metallidurans* | *Nitrosospira multiformis* | *Nitrosospira multiformis* | *Nitrosospira multiformis* |
| *Ralstonia solanacearum* | *Ralstonia solanacearum* | *Ralstonia solanacearum* | *Cupriavidus metallidurans* | *Cupriavidus metallidurans* | *Cupriavidus metallidurans* |
| *Delftia acidovorans* | *Delftia acidovorans* | *Delftia acidovorans* | *Ralstonia solanacearum* | *Ralstonia solanacearum* | *Bacillus cereus* |
| *Cupriavidus necator* | *Cupriavidus necator* | *Cupriavidus necator* | *Delftia acidovorans* | *Cupriavidus necator* | *Ralstonia solanacearum* |
| *Cupriavidus taiwanensis* | *Cupriavidus taiwanensis* | ***Bacillus anthracis*** | *Cupriavidus necator* | *Delftia acidovorans* | *Delftia acidovorans* |
| *Cupriavidus pinatubonensis* | *Hyphomicrobium denitrificans* | *Cupriavidus taiwanensis* | *Rhodococcus erythropolis* | *Cupriavidus taiwanensis* | *Cupriavidus necator* |
| *Stenotrophomonas maltophilia* | *Cupriavidus pinatubonensis* | *Bacillus megaterium* | *Cupriavidus taiwanensis* | *Propionibacterium acnes* | *Cupriavidus taiwanensis* |
| *Hyphomicrobium denitrificans* | *Arthrobacter* sp. | *Rhodococcus erythropolis* | *Hyphomicrobium denitrificans* | *Cupriavidus pinatubonensis* | *Bacillus thuringiensis* |
| uncultured bacterium | *Bacillus megaterium* | *Cupriavidus pinatubonensis* | *Cupriavidus pinatubonensis* | *Hyphomicrobium denitrificans* | *Cupriavidus pinatubonensis* |
| *Pseudomonas aeruginosa* | uncultured bacterium | *Hyphomicrobium denitrificans* | uncultured bacterium | *Pseudomonas fluorescens* | *Hyphomicrobium denitrificans* |
| *Magnetospirillum gryphiswaldense* | *Pseudomonas aeruginosa* | uncultured bacterium | *Bradyrhizobium* sp. BTAi1 | uncultured bacterium | *Arthrobacter* sp. |
| *Pseudomonas fluorescens* | *Stenotrophomonas maltophilia* | *Acidovorax* sp. JS42 | *Bacillus megaterium* | *Bacillus cereus* | *Bacillus weihenstephanensis* |
| *Acidovorax* sp. JS42 | *Bradyrhizobium* sp. BTAi1 | *Pseudomonas aeruginosa* | *Magnetospirillum gryphiswaldense* | *Acidovorax* sp. JS42 | *Stenotrophomonas maltophilia* |
