# Supplementary material for: Detection of Bacillus anthracis DNA in Complex Soil and Air Samples Using Next-Generation Sequencing
Source: PLoS One. 2013 Sep 9;8(9):e73455. doi: 10.1371/journal.pone.0073455 (PMC3767809; doi:10.1371/journal.pone.0073455)
Supplement: Table S4 — The top 15 non-bacterial organisms identified at the lowest B. anthracis genome copy spike-in level in aerosol and soil samples. (DOCX) [file pone.0073455.s005.docx]

**Table S4.** **The top 15 non-bacterial organisms identified at the lowest *B. anthracis* genome copy spike-in level in aerosol and soil samples.**

| **Aerosol samples** | | **Soil samples** | |
| --- | --- | --- | --- |
| **Category** | **Species** | **Category** | **Species** |
| Plants | *Betula nana* | Invertebrates | *Wuchereria bancrofti* |
| Plants | *Alternaria arborescens* | Invertebrates | *Hammondia hammondi* |
| Invertebrates | *Hammondia hammondi* | Plants | *Platanus occidentalis* |
| Plants | *Gibberella zeae* | Plants | *Botryotinia fuckeliana* |
| Plants | *Quercus robur* | Invertebrates | *Acanthamoeba castellanii* |
| Plants | *Aureobasidium pullulans* | Invertebrates | *Dimastigella mimosa* |
| Plants | *Pyrus x bretschneideri* | Plants | *Trichoderma hamatum* |
| Plants | *Pinus taeda* | Rodents | *Mus musculus* |
| Plants | *Botryotinia fuckeliana* | Plants | *Populus trichocarpa* |
| Primates | *Homo sapiens* | Plants | *Triticum aestivum* |
| Invertebrates | *Onchocerca volvulus* | Invertebrates | *Aplysia californica* |
| Plants | *Zea mays* | Plants | *Betula nana* |
| Plants | *Solanum lycopersicum* | Rodents | *Rattus norvegicus* |
| Plants | *Triticum aestivum* | Primates | *Homo sapiens* |
| Plants | *Cladosporium sphaerospermum* | Vertebrates | *Gadus morhua* |
