# Supplementary material for: Detection of Bacillus anthracis DNA in Complex Soil and Air Samples Using Next-Generation Sequencing
Source: PLoS One. 2013 Sep 9;8(9):e73455. doi: 10.1371/journal.pone.0073455 (PMC3767809; doi:10.1371/journal.pone.0073455)
Supplement: Table S5 — Detection ranking of B. anthracis, as determined by total number of mapped reads, following mapping (zero mismatches) of sequence data to all organisms versus bacteria only. (DOCX) [file pone.0073455.s006.docx]

**Table S5. Detection ranking of *B. anthracis*, as determined by total number of mapped reads, following mapping (zero mismatches) of sequence data to all organisms versus bacteria only.**

| **Alignment to all organisms** | | | | | | |
| --- | --- | --- | --- | --- | --- | --- |
| *B. anthracis* genome equivalents | 1 | 10 | 100 | 1,000 | 10,000 | 100,000 |
| Aerosol ranking of *B. anthracis* | 47 | 13 | 5 | 2 | 1 | 1 |
| Soil ranking of *B. anthracis* | 228 | 36 | 5 | 1 | 1 | 1 |
|  |  |  |  |  |  |  |
| **Alignment to bacteria only** | | | | | | |
| *B. anthracis* genome equivalents | 1 | 10 | 100 | 1,000 | 10,000 | 100,000 |
| Aerosol ranking of *B. anthracis* | 20 | 8 | 2 | 1 | 1 | 1 |
| Soil ranking of *B. anthracis* | 99 | 21 | 4 | 1 | 1 | 1 |
