# Supplementary material for: Detection of Bacillus anthracis DNA in Complex Soil and Air Samples Using Next-Generation Sequencing
Source: PLoS One. 2013 Sep 9;8(9):e73455. doi: 10.1371/journal.pone.0073455 (PMC3767809; doi:10.1371/journal.pone.0073455)
Supplement: Table S6 — Mapping of 454 sequencing reads to the GenBank reference database. The top 15 taxonomic IDs observed in 454 sequence data from each B. anthracis-spiked environmental sample were compiled into a union set of species most prominently observed. Each species is listed with its corresponding normalized number of mapped reads (normalized to total reads for each given sample). The species identified by 454 sequencing in both aerosol and soil samples are shown. (DOCX) [file pone.0073455.s007.docx]

**Table S6. Mapping of 454 sequencing reads to the GenBank reference database.** The top 15 taxonomic IDs observed in 454 sequence data from each *B. anthracis*-spiked environmental sample were compiled into a union set of species most prominently observed. Each species is listed with its corresponding normalized number of mapped reads (normalized to total reads for each given sample). The species identified by 454 sequencing in both aerosol and soil samples are shown.

|  | ***B. anthracis* genome equivalents** | | | |
| --- | --- | --- | --- | --- |
| **Organism** | **0** | **1** | **10** | **100** |
| ***Aerosol sample normalized mapped reads*** | | | | |
| *Agrobacterium radiobacter* | 0.00E+00 | 1.74E-03 | 0.00E+00 | 6.91E-06 |
| *Alternaria alternata* | 4.55E-04 | 2.78E-04 | 1.69E-03 | 1.02E-03 |
| *Artemisia annua* | 2.69E-04 | 1.60E-03 | 4.07E-05 | 1.11E-04 |
| *Aspergillus niger* | 5.51E-04 | 1.12E-03 | 3.93E-04 | 4.18E-04 |
| *Bacillus anthracis* | 0.00E+00 | 7.95E-04 | 7.53E-03 | 6.66E-02 |
| *Bacillus cereus* | 8.67E-06 | 2.10E-04 | 1.93E-03 | 1.31E-02 |
| *Botryotinia fuckeliana* | 1.01E-03 | 4.37E-04 | 5.83E-04 | 9.95E-04 |
| *Castanea mollissima* | 2.72E-03 | 7.61E-04 | 6.92E-04 | 4.97E-04 |
| *Delftia acidovorans* | 2.13E-03 | 4.42E-03 | 1.25E-02 | 5.20E-03 |
| *Gibberella zeae* | 1.14E-03 | 1.91E-03 | 1.11E-03 | 1.03E-03 |
| *Klebsiella pneumoniae* | 1.73E-05 | 0.00E+00 | 1.36E-05 | 9.84E-04 |
| *Leptosphaeria maculans* | 2.29E-03 | 2.96E-03 | 2.49E-03 | 1.79E-03 |
| *Microbotryum violaceum* | 1.56E-04 | 1.18E-03 | 4.07E-05 | 1.62E-04 |
| *Nectria haematococca* | 7.81E-04 | 9.55E-04 | 7.32E-04 | 6.35E-04 |
| *Neurospora crassa* | 7.55E-04 | 1.12E-03 | 4.88E-04 | 8.56E-04 |
| *Oryza sativa* | 1.10E-03 | 1.11E-03 | 6.10E-04 | 7.94E-04 |
| *Pantoea vagans* | 8.80E-04 | 6.88E-03 | 1.90E-04 | 1.27E-03 |
| *Penicillium chrysogenum* | 1.49E-03 | 1.12E-03 | 9.36E-04 | 1.41E-03 |
| *Phaeosphaeria nodorum* | 3.31E-03 | 4.10E-03 | 2.62E-03 | 3.15E-03 |
| *Pinus taeda* | 9.36E-03 | 5.01E-03 | 3.67E-03 | 3.19E-03 |
| *Podospora anserina* | 4.99E-04 | 5.34E-04 | 7.73E-04 | 4.56E-04 |
| *Pyrenophora tritici-repentis* | 5.05E-03 | 9.28E-03 | 4.50E-03 | 5.07E-03 |
| *Ralstonia eutropha* | 1.18E-03 | 0.00E+00 | 0.00E+00 | 0.00E+00 |
| *Ralstonia pickettii* | 2.56E-03 | 5.89E-05 | 5.42E-05 | 4.14E-05 |
| *Ralstonia solanacearum* | 8.98E-04 | 0.00E+00 | 0.00E+00 | 0.00E+00 |
| *Stenotrophomonas maltophilia* | 4.34E-05 | 1.35E-04 | 5.02E-03 | 4.03E-03 |
| *Vitis vinifera* | 1.32E-03 | 4.96E-04 | 8.54E-04 | 4.70E-04 |
| ***Soil sample normalized mapped reads*** | | | | |
| *Acidobacterium capsulatum* | 1.68E-04 | 6.70E-05 | 3.12E-05 | 1.99E-05 |
| *Arthrobacter* sp. | 1.05E-04 | 2.44E-04 | 1.09E-04 | 7.98E-05 |
| *Bacillus anthracis* | 0.00E+00 | 1.53E-04 | 1.61E-03 | 2.63E-02 |
| *Bacillus cereus* | 0.00E+00 | 1.44E-05 | 2.57E-04 | 5.76E-03 |
| *Bacillus megaterium* | 1.47E-04 | 1.92E-05 | 7.80E-06 | 3.49E-04 |
| *Cloning vector* | 2.10E-05 | 5.27E-05 | 8.58E-05 | 0.00E+00 |
| *Crepidula fornicata* | 3.92E-04 | 7.18E-05 | 7.80E-05 | 0.00E+00 |
| *Cupriavidus metallidurans* | 5.33E-04 | 0.00E+00 | 0.00E+00 | 0.00E+00 |
| *Cupriavidus taiwanensis* | 4.41E-04 | 0.00E+00 | 0.00E+00 | 0.00E+00 |
| *Danio rerio* | 5.36E-03 | 7.61E-04 | 9.75E-04 | 4.29E-04 |
| *Delftia acidovorans* | 7.01E-05 | 1.92E-05 | 1.17E-04 | 6.98E-05 |
| *Dictyostelium discoideum* | 3.08E-04 | 4.79E-05 | 4.68E-05 | 3.99E-05 |
| *Dimastigella mimosa* | 3.93E-03 | 6.25E-03 | 8.12E-03 | 7.54E-03 |
| *Haliangium ochraceum* | 0.00E+00 | 0.00E+00 | 0.00E+00 | 1.10E-04 |
| *Homo sapiens* | 2.73E-04 | 8.14E-05 | 1.09E-04 | 9.97E-05 |
| *Monosiga brevicollis* | 3.36E-04 | 6.70E-05 | 7.80E-05 | 0.00E+00 |
| *Mus musculus* | 3.99E-03 | 6.18E-04 | 8.35E-04 | 4.79E-04 |
| *Mus spretus* | 1.12E-04 | 9.58E-06 | 8.58E-05 | 9.97E-06 |
| *Nitrosospira multiformis* | 3.97E-03 | 1.29E-03 | 1.15E-03 | 7.48E-04 |
| *Oncorhynchus mykiss* | 5.47E-04 | 7.66E-05 | 1.56E-04 | 3.99E-05 |
| *Platanus occidentalis* | 2.31E-04 | 6.70E-05 | 1.33E-04 | 9.97E-05 |
| *Propionibacterium acnes* | 0.00E+00 | 0.00E+00 | 0.00E+00 | 2.49E-04 |
| *Pseudomonas fluorescens* | 2.87E-04 | 1.82E-04 | 2.57E-04 | 1.99E-05 |
| *Ralstonia eutropha* | 7.22E-04 | 0.00E+00 | 0.00E+00 | 0.00E+00 |
| *Ralstonia pickettii* | 2.77E-03 | 0.00E+00 | 0.00E+00 | 0.00E+00 |
| *Ralstonia solanacearum* | 1.02E-03 | 0.00E+00 | 7.80E-06 | 0.00E+00 |
| *Rattus norvegicus* | 4.98E-04 | 6.70E-05 | 6.24E-05 | 1.79E-04 |
| *Salmo salar* | 3.22E-04 | 3.83E-05 | 3.90E-05 | 1.99E-05 |
| *Sphingomonas wittichii* | 1.40E-05 | 9.58E-06 | 0.00E+00 | 1.20E-04 |
| *Spirosoma linguale* | 0.00E+00 | 0.00E+00 | 0.00E+00 | 5.58E-03 |
| *Uncultured bacterium* | 1.75E-04 | 4.31E-05 | 9.36E-05 | 2.19E-04 |
| *Vitis vinifera* | 1.54E-04 | 1.05E-04 | 7.02E-05 | 1.99E-05 |
