# Supplementary material for: Detection of Bacillus anthracis DNA in Complex Soil and Air Samples Using Next-Generation Sequencing
Source: PLoS One. 2013 Sep 9;8(9):e73455. doi: 10.1371/journal.pone.0073455 (PMC3767809; doi:10.1371/journal.pone.0073455)
Supplement: Table S7 — Summary of microbial sequences represented on the census array. (DOCX) [file pone.0073455.s008.docx]

**Table S7. Summary of microbial sequences represented on the census array.**

| Number of Targets | Viral | Bacterial |
| --- | --- | --- |
| Families | 80 | 274 |
| Groups without family classification | 48 | 65 |
| Species with complete genome, plasmid, or segment data | 2530 | 1290 |
| Species with sequence data, including sequence fragments | 5719 | 14765 |
| Sequences classified as to family | 171264 | 728467 |
| Sequences unclassified as to family | 6996 | 56251 |
| Complete genomes, segments, or plasmids | 55803 | 4122 |
