# Supplementary material for: Detection of Bacillus anthracis DNA in Complex Soil and Air Samples Using Next-Generation Sequencing
Source: PLoS One. 2013 Sep 9;8(9):e73455. doi: 10.1371/journal.pone.0073455 (PMC3767809; doi:10.1371/journal.pone.0073455)
Supplement: Table S8 — Top hit organisms detected by the census array in B. anthracis-spiked aerosol and soil samples. (DOCX) [file pone.0073455.s009.docx]

**Table S8. Top hit organisms detected by the census array in *B. anthracis*-spiked aerosol and soil samples.**

| *B. anthracis* genome equivalents | | | | | |
| --- | --- | --- | --- | --- | --- |
| **1** | **10** | **100** | **1,000** | **10,000** | **100,000** |
| **Aerosol background** | | | | | |
| *Magnetospirillum magnetotacticum* | *Magnetospirillum magnetotacticum* | ***Bacillus anthracis*** | ***Bacillus anthracis*** | ***Bacillus anthracis*** | ***Bacillus anthracis*** |
| *Thioalkalivibrio* sp. | ***Bacillus anthracis**** | *Bacillus megaterium* | *Magnetospirillum magnetotacticum* | *Thioalkalivibrio* sp. | *Thioalkalivibrio* sp. |
| *Bacillus megaterium* | *Thioalkalivibrio* sp. | *Magnetospirillum magnetotacticum* | *Alkalilimnicola ehrlichei* | *Ralstonia pickettii* | *Ralstonia metallidurans* |
| *Ralstonia pickettii* | *Ralstonia pickettii* | *Alkalilimnicola ehrlichei* | *Bradyrhizobium* sp. | *Ralstonia metallidurans* | *Stackebrandtia nassauensis* |
| *Ralstonia metallidurans* | *Ralstonia metallidurans* | *Tolumonas auensis* | *Geodermatophilus obscurus* | *Catenulispora acidiphila* | *Catenulispora acidiphila* |
| *Saccharopolyspora erythraea* | *Serratia proteamaculans* | *Nakamurella multipartita* | *Vibrio splendidus* | *Thermomonospora curvata* | *Dickeya zeae* |
| *Sodalis glossinidius* | *Thermomonospora curvata* | *Bradyrhizobium* sp. | *Haliangium ochraceum* | *Haliangium ochraceum* | Plantago asiatica mosaic virus |
| *Salmonella enterica* | *Bradyrhizobium* sp. | *Verminephrobacter eiseniae* | *Klebsiella pneumoniae* | *Buchnera aphidicola* | *Variovorax paradoxus* |
| *Haliangium ochraceum* | *Haliangium ochraceum* |  |  | *Klebsiella pneumoniae* | *Bovine adenovirus B* |
| *Delftia acidovorans* | *Catenulispora acidiphila* |  |  | *Plantago asiatica* |  |
| **Soil background** | | | | | |
| *Magnetospirillum magnetotacticum* | *Magnetospirillum magnetotacticum* | ***Bacillus anthracis**** | ***Bacillus anthracis*** | ***Bacillus anthracis*** | ***Bacillus anthracis*** |
| *Alkalilimnicola ehrlichei* | *Anoxybacillus flavithermus* | *Methylococcus capsulatus* | *Thioalkalivibrio* sp. | *Methylococcus capsulatus* | *Halothiobacillus neapolitanus* |
| *Bacillus megaterium* | *Tolumonas auensis* | *Kineococcus radiotolerans* | *Kineococcus radiotolerans* | *Kineococcus radiotolerans* | *Corynebacterium matruchotii* |
| *Kineococcus radiotolerans* | *Nakamurella multipartita* | *Nitrosospira multiformis* | *Ralstonia eutropha* | *Ralstonia pickettii* | *Ralstonia pickettii* |
| *Photobacterium profundum* | *Psychroflexus torquis* | *Ralstonia metallidurans* | *Ralstonia pickettii* | *Ralstonia metallidurans* | *Ralstonia metallidurans* |
| *Chitinophaga pinensis* | *Nitrosospira multiformis* | *Ralstonia pickettii* | *Tolumonas auensis* | Ostreococcus virus | Citrus exocortis viroid |
| *Nitrosospira multiformis* | *Actinosynnema mirum* | *Vibrio fischeri* | *Methylobacterium radiotolerans* | *Photobacterium profundum* | Ostreococcus virus |
| Ostreococcus virus | *Acidobacterium capsulatum* | Ostreococcus virus | *Catenulispora acidiphila* | *Aeromonas bestiarum* |  |
| *Arthrobacter* sp. | *Methylibium petroleiphilum* | Citrus exocortis viroid | *Haliangium ochraceum* |  |  |
| Asparagus virus 3 | Asparagus virus 3 |  |  |  |  |

******B. anthracis* detected in only one of two replicates.
