# Supplementary material for: Detection of Bacillus anthracis DNA in Complex Soil and Air Samples Using Next-Generation Sequencing
Source: PLoS One. 2013 Sep 9;8(9):e73455. doi: 10.1371/journal.pone.0073455 (PMC3767809; doi:10.1371/journal.pone.0073455)
Supplement: Table S9 — Census array probe sequence matches to metagenomic sequence data from soil samples for bacterial species not anticipated in conventional environmental samples. (DOCX) [file pone.0073455.s010.docx]

**Table S9. Census array probe sequence matches to metagenomic sequence data from soil samples for bacterial species not anticipated in conventional environmental samples.**

| **Organism** | **Soil sample** | **# Unique probes with matches to metagenome** | **# Unique reads with matches to positive organism probes** |
| --- | --- | --- | --- |
| *Tolumonas auensis* | Ba 1 copy | 88 | 905 |
|  | Ba 10 copies | 114 | 1337 |
|  | Ba 100 copies | 100 | 915 |
|  | Ba 1000 copies | 103 | 1059 |
|  | Ba 10000 copies | 98 | 1237 |
|  | Ba 100000 copies | 45 | 1247 |
| *Thioalkalivibrio* | Ba 1 copy | 13 | 258 |
|  | Ba 10 copies | 13 | 548 |
|  | Ba 100 copies | 13 | 315 |
|  | Ba 1000 copies | 11 | 396 |
|  | Ba 10000 copies | 12 | 393 |
|  | Ba 100000 copies | 5 | 507 |
| *Psychroflexus torquis* | Ba 1 copy | 52 | 2804 |
|  | Ba 10 copies | 54 | 3930 |
|  | Ba 100 copies | 53 | 2642 |
|  | Ba 1000 copies | 58 | 3580 |
|  | Ba 10000 copies | 54 | 4692 |
|  | Ba 100000 copies | 47 | 6298 |
| *Haliangium ochraceum* | Ba 1 copy | 1 | 1 |
|  | Ba 10 copies | 2 | 2 |
|  | Ba 100 copies | 4 | 5 |
|  | Ba 1000 copies | 2 | 2 |
|  | Ba 10000 copies | 2 | 2 |
|  | Ba 100000 copies | 1 | 1 |
| *Photobacterium profundum* | Ba 1 copy | 55 | 138 |
|  | Ba 10 copies | 87 | 184 |
|  | Ba 100 copies | 61 | 143 |
|  | Ba 1000 copies | 72 | 264 |
|  | Ba 10000 copies | 65 | 660 |
|  | Ba 100000 copies | 35 | 869 |
